# Development and validation of a novel prognostic signature based on m6A/m5C/m1A-related genes in hepatocellular carcinoma

**DOI:** 10.1186/s12920-023-01611-x

**Published:** 2023-07-31

**Authors:** Yu Xiao, Jinluan Li, Junxin Wu

**Affiliations:** Department of Radiation Oncology, Clinical Oncology School of Fujian Medical University, Fujian Cancer Hospital, Fuzhou, 350014 China

**Keywords:** m6A, m5C, m1A, Hepatocellular cancer, Prognosis

## Abstract

**Background:**

RNA methylation modification plays an important role in cancers. This study sought to examine the association between m6A/m5C/m1A-related genes and hepatocellular carcinoma (HCC).

**Methods:**

Gene expression and clinical data of HCC patients were obtained from the TCGA database. Unsupervised consensus clustering was performed according to the expression of m6A/m5C/m1A-related genes in HCC. The relationships among prognosis, clinicopathological features and molecular subtypes were analyzed. Least absolute shrinkage and selection operator (LASSO) regression analysis was used to establish the m6A/m5C/m1A-related gene prognostic signature. Furthermore, the prognostic signature was validated based on the ICGC dataset. RT‒qPCR was used to detect the expression of the model genes in HCC. Clinicopathological features, functional enrichment, gene mutations, immune cell infiltration, and immunotherapy response in different risk groups were analyzed. A nomogram based on risk score and stage was constructed to predict HCC patient prognosis.

**Results:**

Two m6A/m5C/m1A-related molecular subtypes were identified in HCC, and the prognosis of cluster C1 was worse than that of cluster C2 (*p* < 0.001). Highly expressed genes in cluster C1 are significantly correlated with G3-4, T3-4, stage III-IV (*p* < 0.05). An m6A/m5C/m1A-related prognostic signature was established and validated. The RT‒qPCR results showed that the risk signature genes were significantly upregulated in liver cancer tissue (*p* < 0.05). The prognosis of HCC patients in the high-risk group was worse than that of those in the low-risk group (*p* < 0.05). Multivariate Cox analysis indicated that the risk score was an independent factor predicting prognosis in HCC patients. ssGSEA revealed that the risk score correlated with the tumor immune microenvironment in HCC. Gene mutation analysis showed that the tumor mutation burden of patients in the high-risk group was much higher (*p* < 0.05), and the prognosis of HCC patients with high risk scores and high mutation burden was the worst (*p* = 0.007). A nomogram combining risk scores with clinicopathological features showed performed well in predicting HCC prognosis.

**Conclusions:**

The m6A/m5C/m1A-related genes could predict the prognosis and tumor microenvironment features of HCC and can be important biomarkers relevant to the immunotherapy response.

**Supplementary Information:**

The online version contains supplementary material available at 10.1186/s12920-023-01611-x.

## Background

Hepatocellular carcinoma (HCC) is one of the most common malignant tumors in the world. In 2020, there were approximately 906,000 newly diagnosed cases of HCC worldwide and 830,000 deaths from HCC, making it the sixth most common cancer and the third leading cause of cancer-related death [1, 2]. HCC has insidious, rapid onset and an extremely high degree of malignancy [3]. The 5-year survival rate is less than 18% [1, 4], which seriously affects public health. Considering the limitations of HCC treatment, new therapeutic targets are needed to improve the prognosis of HCC patients. Therefore, it is urgent to find a prognosis-related diagnostic model that could provide new directions for developing feasible targeted therapy approaches and improving the survival and prognosis of patients.

Epigenetic modifications include chemical modifications of DNA, RNA and proteins characterized by altered gene expression and function without any changes in the gene sequence. In addition to well-established DNA and protein epigenetic modifications, reversible RNA methylation has led to the third wave of studies in the epigenetic field. The main forms of RNA methylation are N6-methyladenosine (m6A), 5-methylcytosine (m5C) and N1-methyladenosine (m1A). m6A is the most abundant internal RNA modification in eukaryotic cells.

A large number of scholars have previously conducted research regarding the mechanism of m6A/m5C/m1A-related genes in HCC. LY6/PLAUR domain 1 (LYPD1) can promote tumorigenesis, ALKB Homolog 5 (ALKBH5) mediated m6A demethylation leads to posttranscriptional repression of LYPD1, and dysregulation of the ALKBH5/LYPD1 axis leads to HCC progression [5]. Wang et al. [6] found that high expression level of circ-KIAA1429 in hepatoma cells and KIAA1429 acts as an oncogene to promote HCC invasion and migration by altering the methylation of m6A in Inhibitor of DNA binding 2(ID2) and GATA-binding protein(GATA3) mRNAs [7]. YTH N6-methyladenosine RNA binding protein F3 (YTHDF3) can increase the stability of zinc finger e-box binding homeobox 1 (Zeb1) mRNA and participate in the occurrence and development of liver cancer [6]. Methyltransferase-like 3 (METTL3) and Methyltransferase-like 14 (METTL14) are the two core molecules. However, METTL3 and METTL14 exert opposing regulatory roles in HCC [8]. Hepatitis B virus X-interacting protein (HBXIP) interference suppressed the malignant behavior of HCC and suppressed the Warburg effect in HCC cells. METTL3 is upregulated in HCC tissues and positively regulated by HBXIP. HBXIP-mediated METTL3 promotes metabolic reprogramming and proliferation, invasion and metastasis of HCC cells [9]. The expression of ubiquitin-specific peptidase 48 (USP48) is significantly reduced in HCC, and methyltransferase-like 14 (Mettl14)-induced m6A modification is involved in the regulation of USP48 in HCC by maintaining USP48 mRNA stability, thereby inhibiting the development of HCC [10]. YTH N6-methyladenosine RNA binding protein 1 (YTHDF1) can promote the occurrence and development of liver cancer through various pathways. Significant overexpression of YTHDF1 in HCC tissues is associated with a poor prognosis, and YTHDF1 deficiency inhibits HCC autophagy, growth and metastasis [11]. YTHDF1 can accelerate translational export of FZD5 mRNA in an m6A-dependent manner and function as an oncogene through the WNT/β-catenin pathway [12]. The m6A demethylase FTO promotes hepatocellular carcinoma tumorigenesis by mediating pyruvate kinase M2 (PKM2) demethylation [13]. NOP2/Sun RNA methyltransferase 2 (NSUN2) is an RNA methyltransferase responsible for m5C modification of multiple RNAs. The H19 lncRNA is a specific target of NSUN2 modifiers. m5C-modified H19 lncRNA may promote tumorigenesis and development by recruiting the G3BP1 protein [14]. NOP2/Sun RNA methyltransferase 4 (NSUN4) is significantly upregulated in tissues and cells of HCC patients and is closely related to the occurrence and development of HCC [15]. Aly/REF export Factor (ALYREF) is significantly upregulated in liver cancer tissues and liver cancer cell lines and is significantly associated with poor prognosis in HCC [16]. TRNA methyltransferase 6 non-catalytic subunit (TRMT6) and TRNA methyltransferase 61A (TRMT61A) form the m1A methyltransferase complex, which is highly expressed in advanced HCC tumors and negatively correlates with HCC survival [17]. Emerging reports confirm that dysregulation of RNA methylation contributes to a variety of human diseases, particularly hepatocellular carcinoma. However, no study has comprehensively analyzed the relationship between these several forms of RNA methylation and HCC.

Therefore, based on the existing research on RNA methylation in HCC, we collected m6A/m5C/m1A-related genes that are closely correlated with the occurrence and development of HCC. We aimed to explore the relationship between the expression levels of m6A/m5C/m1A-related genes and prognosis of HCC patients. Furthermore, we tried to construct and validate a m6A/m5C/m1A-related gene prognostic signature and explore its relationship with the clinicopathological features, immune microenvironment and immunotherapy of HCC, in order to provide individualized strategies for clinical treatment of HCC.

## Methods

### Data sources

We extracted 10 m6A/m5C/ m1A-related genes that are closely related to the occurrence and development of HCC, and the information on m6A/m5C/m1A-related genes were listed in Table 1. HCC RNA-sequencing (RNA-seq) data and clinical data (Table S1) were downloaded from the TCGA database (https://portal.gdc.cancer.gov/). Moreover, we downloaded HCC RNA-sequencing (RNA-seq) data and clinical data (Table S2) from the ICGC database (https://dcc.icgc.org/) as external validation data.

**Table 1:** Information of m6A/m5C/m1A-related genes in HCC

Gene symbol	ENSG	Chromosome	ChromStart	ChromEnd	Modification type
NSUN4	ENSG00000117481	chr1	46340177	46365152	m5C
METTL14	ENSG00000145388	chr4	118685368	118715433	m6A
NSUN2	ENSG00000037474	chr5	6599239	6633291	m5C
YTHDF3	ENSG00000185728	chr8	63168553	63212786	m6A
METTL3	ENSG00000165819	chr14	21498133	21511375	m6A
FTO	ENSG00000140718	chr16	53703963	54121941	m6A
ALKBH5	ENSG00000091542	chr17	18183078	18209954	m6A
ALYREF	ENSG00000183684	chr17	81887844	81891586	m5C
TRMT6	ENSG00000089195	chr20	5937235	5950558	m1A
YTHDF1	ENSG00000149658	chr20	63195429	63216234	m6A

### Copy number variation and differential expression analysis of m6A/m5C/m1A-related genes in HCC

The copy number variation frequency information of TCGA HCC samples were downloaded from the UCSC Xena website (https://xena.ucsc.edu/). We used the “limma” package to distinguish differentially expressed genes (DEGs) with *p* values < 0.05. Then, we constructed a PPI network associated with m6A/m5C/m1A genes using the STRING website (https://string-db.org/) for analysis of interacting genes. Furthermore, the correlation analysis based on each DEG was performed using the R software "reshape2" package and "igraph" packages.

### The m6A/m5C/m1A-related molecular subtypes identification

We used the R software "ConsensusClusterPlus" and "limma" packages to classify HCC patients according to the expression of m6A/m5C/m1A-related genes. Using the R software "survival" package, we analyzed the prognosis of patients with different molecular subtypes. Furthermore, the "limma" package was used to analyze the relationship between molecular subtypes and clinicopathological characteristics.

### Establishment and validation of m6A/m5C/m1A-related gene prognostic signature

The R software "survival" R package was used to perform univariate Cox analysis on HCC patients in TCGA to study the effect of m6A/m5C/m1A-related genes on prognosis. Then, using the R software package "glmnet", the prognostic genes screened by univariate Cox analysis were used to construct a prognostic signature by LASSO Cox regression analysis. An individualized risk score was obtained based on the expression level of the prognostic genes and the estimated regression coefficients in LASSO Cox regression analysis. The risk score for each HCC patient was calculated by the following formula:

$$\mathrm{Risk}\;\mathrm{Score}=\sum\nolimits_{\mathrm i=1}^{\mathrm n}\left(\mathrm{Expi}\ast\mathrm{Coei}\right)$$

HCC patients were divided into high- and low-risk groups according to the median risk score, and Kaplan‒Meier analysis was performed to compare 1-, 2-, and 3-year overall survival. The R packages "survival", "survminer" and "time ROC" were used to conduct a receiver operating characteristic (ROC) curve analysis across time periods of 1-, 2-, and 3- years. The distribution and survival of patients are displayed by the "bioRiskPlot" function according to the risk score. Principal component analysis (PCA) was used to assess the samples in each risk group based on their similarities and differences. Patients in the ICGC cohort were separated into low- and high-risk groups based on the median risk score from the TCGA cohort. Then, the ICGC cohort was used to validate the prognostic signature.

### Prognostic signature genes mRNA expression analysis via RT‒qPCR

Tissue microarray of human liver cancer and paired adjacent normal tissues (MecDNA-HLivH060PG02) was purchased from Shanghai Outdo Biotech Company (Shanghai, China). The study was approved by the Ethics Committee of Shanghai Outdo Biotech Company. The tissue microarray contains 30 paired cancer and non-cancerous liver tissues. To validate gene expression, quantitative real-time PCR (qRT-PCR) analyses were performed. β-Actin served as an internal reference. Primer sequences are listed in Table S3.

### Identification of independent prognostic factors

The R software “survival” package was used for univariate and multivariate analyses. In this analysis, variable factors included age, gender, grade, stage and risk score. Furthermore, we used the “limma” and “ggpubr” packages to analyze the relationship between risk scores and clinicopathological features.

### Functional enrichment and immune microenvironment analysis

HCC patients were divided into two groups according to the median risk score. DEGs were screened between the high- and low-risk groups (FDR < 0.05, |log2FC|≥ 1). On this basis, the "clusterProfiler" software package was used for GO and KEGG analysis [18–20]. The "GSVA" package was used for single-sample gene set enrichment analysis (ssGSEA) to calculate the scores of infiltrating immune cells and the activity of immune-related pathways in high- and low-risk groups of HCC patients. We further used the R software "limma" package to analyze the differential expression of immune checkpoints between high and low risk groups.

### Gene mutation analysis

We further downloaded the HCC simple nucleotide variation data from the TCGA GDC database (https://portal.gdc.cancer.gov/) and then used the R software "maftools" to analyze the gene mutations of the high- and low-risk groups and draw a waterfall diagram. Furthermore, we analyzed the relationship between risk group and tumor mutation burden.

### Development and validation of a nomogram

We used the "rms" package to construct a prediction nomogram based on the clinical features and prognostic signature risk score. In the nomogram scoring approach, each variable was given a score, and the total score was computed by adding the scores for all variables in each sample. The calibration plots of the nomogram were used to highlight the predictive value by comparing the predictions vs. actual outcomes in terms of 1-, 2-, and 3-year survival. Area under the curve of ROC was used to evaluate the ability of different prognostic factors to predict the 1-, 2-, and 3-year survival of HCC patients.

### Statistical analyses

R version 4.1.0 was used for all statistical analyses. The level of statistical significance was set at *p* < 0.05.

## Results

### The landscape of m6A/m5C/m1A-related genes in HCC

**Fig. 1:**
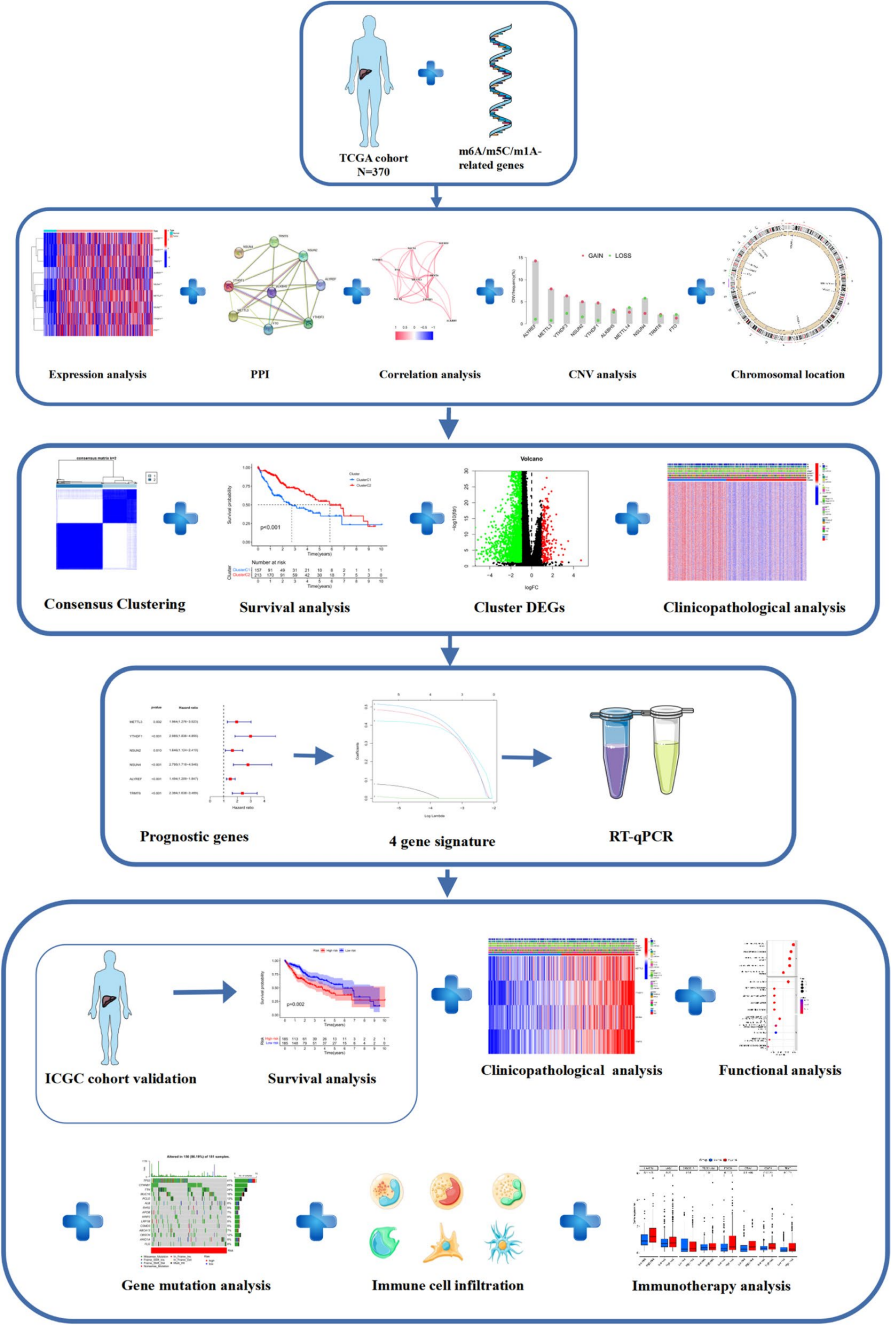
Flow chart of this study

**Fig. 2:**
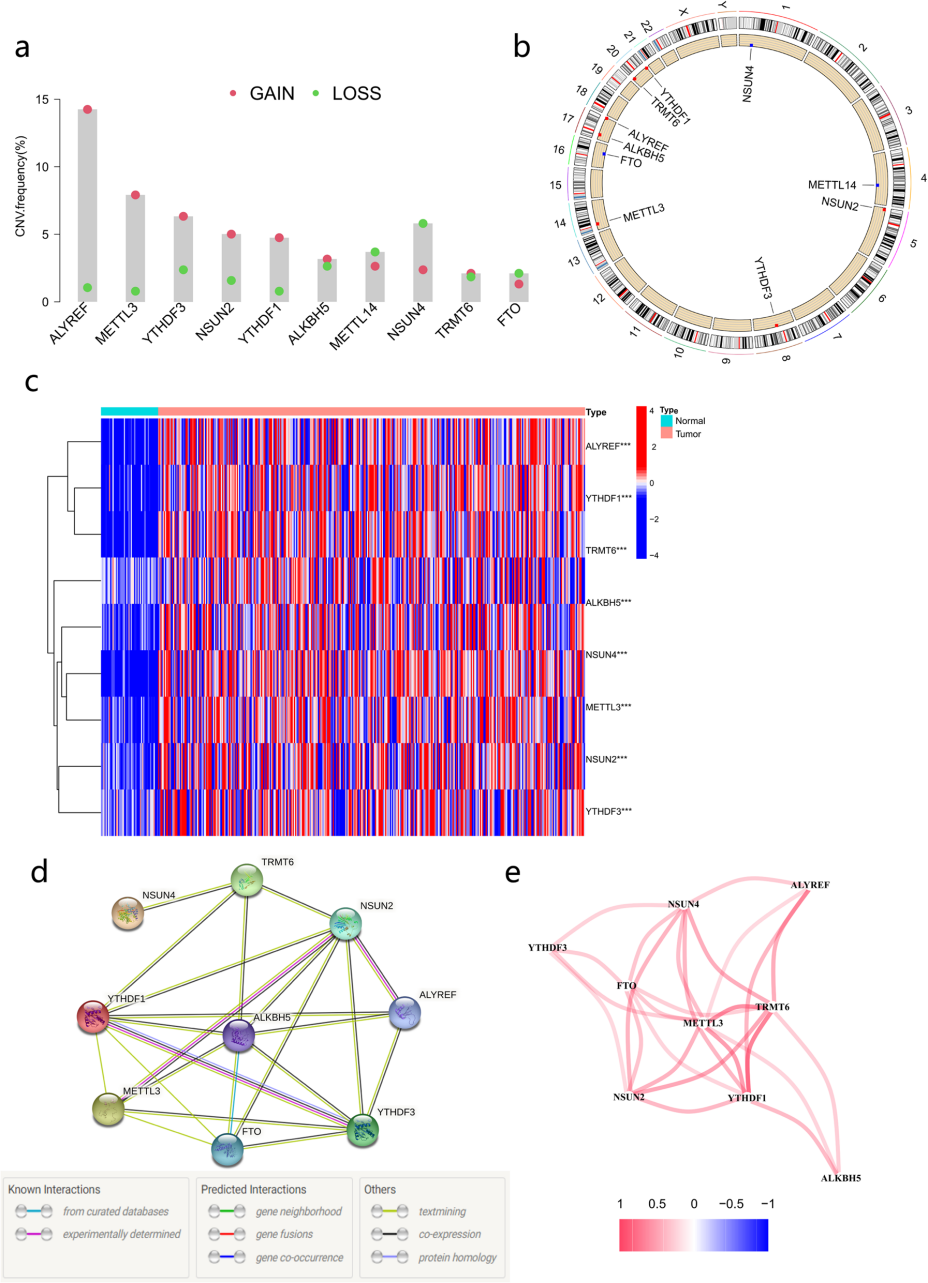
Expression and interaction of m6A/m5C/m1A-related genes. **a** Frequencies of CNV gain, loss, and no CNV among m6A/m5C/m1A-related genes. **b** Locations of CNV alterations in m6A/m5C/m1A-related genes on 23 chromosomes. **c** Heatmap of the genes concerning m6A/m5C/m1A between HCC tissues and normal tissues. **d** The PPI network indicated the interaction of genes concerning m6A/m5C/m1A (interaction score = 0.4). **e** Relevant network of the genes concerning m6A/m5C/m1A. CNV, copy number variation; HCC, hepatocellular carcinoma. PPI, protein–protein interaction. *p* values: *p < 0.05, ** *p* < 0.01, and *** *P* < 0.001

Figure 1 illustrates our study process. We investigated copy number variation (CNV) in m6A/m5C/m1A-related genes and discovered that CNV was prevalent in all genes. ALKBH5, METTL3, YTHDF3, YTHDF1, FTO, NSUN2 and ALYREF exhibited noticeable acquired CNV, but FTO, NSUN4, and METTL14 had reduced CNV (Fig. 2a). Figure 2b depicts the position of the m6A/m5C/m1A-related genes on their respective chromosomes. Based on TCGA data including 376 HCC and 50 normal liver tissues, we compared the expression levels of 10 m6A/m5C/m1A-related genes and finally found 9 DEGs (*p* < 0.05). ALKBH5, METTL3, YTHDF3, YTHDF1, FTO, NSUN2, NSUN4, ALYREF and TRMT6 were significantly upregulated in HCC tissues vs. normal tissues (Fig. 2c). The PPI network revealed the interaction of m6A/m5C/m1A-related genes (Fig. 2d). Figure 2e shown the correlation network of m6A/m5C/m1A-related genes.

### Cluster analysis based on the expression of m6A/m5C/m1A-related genes in HCC

**Fig. 3:**
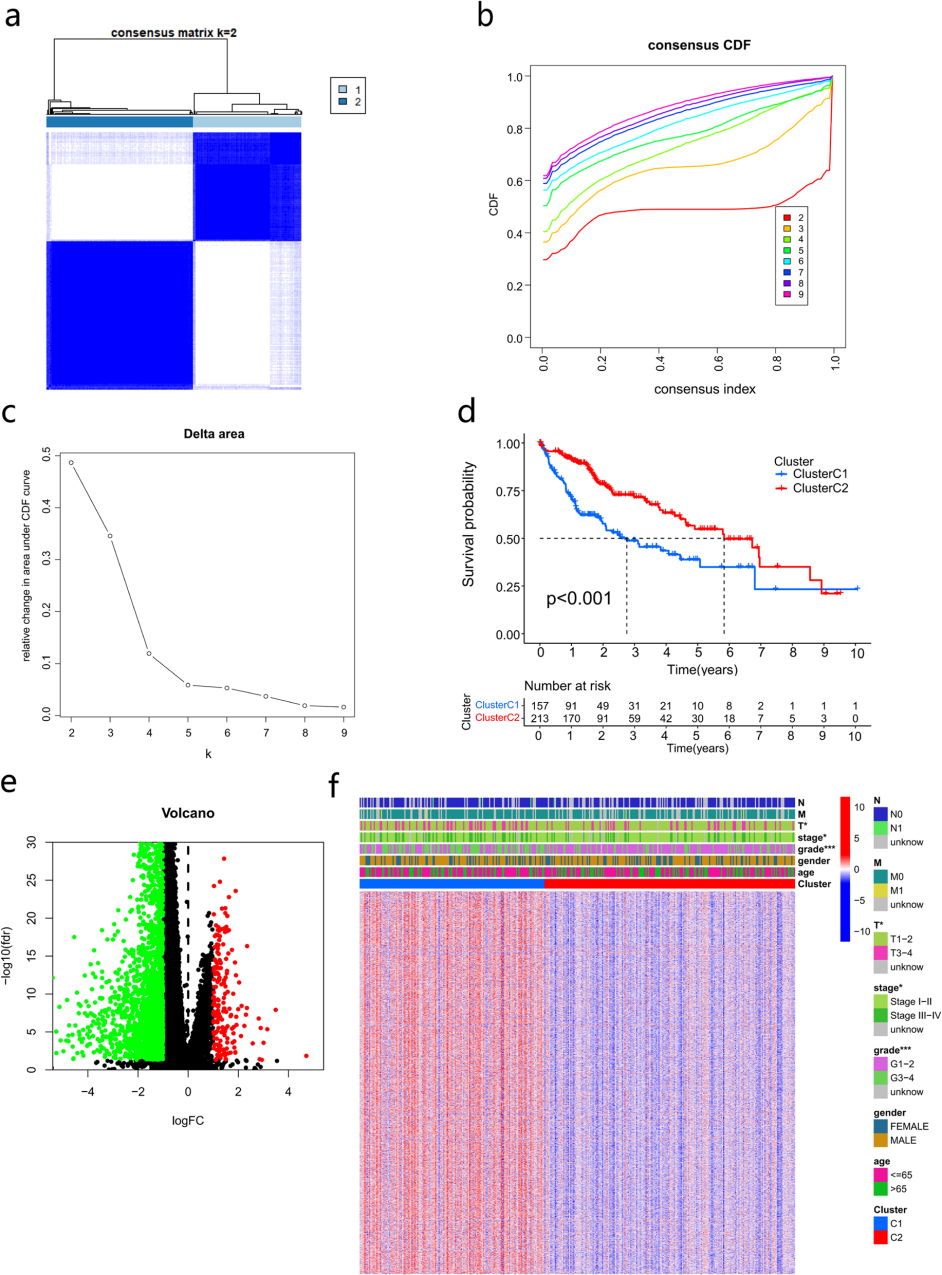
Identification of m6A/m5C/m1A-related subtypes. **a** A total of 370 HCC patients were classified into two subtypes based on consensus cluster analysis. **b** CDF for k = 2 to 9. **c** Relative changes in the areas under the CDF curve for k = 2 to 9. **d** OS of different m6A/m5C/m1A-related subtypes. **e** Identification of DEGs between the two subtypes. **f** The relationship between m6A/m5C/m1A-related subtypes and clinicopathological features in HCC. CDF, cumulative distribution function; OS, overall survival; DEGs, differentially expressed genes; HCC, hepatocellular carcinoma

To better understand the expression characteristics of m6A/m5C/m1A-related genes in HCC, we used a consensus clustering method to classify HCC patients based on the expression levels of 10 m6A/m5C/m1A-related genes. Our results indicated that k = 2 appeared to be the best choice to classify all HCC patients into the cluster C1 (*n* = 157) and cluster C2 (*n* = 213) (Fig. 3a-c). The Kaplan‒Meier curve revealed that the overall survival/prognosis of the cluster C2 was significantly better than that of the cluster C1 (*p* < 0.001) (Fig. 3d). DEGs of the two molecular subtypes were identified (Fig. 3e). According to the DEGs expression of subtypes, we analyzed the correlation between molecular subtypes and clinicopathological features. Highly expressed genes in cluster C1 are significantly correlated with G3-4, T3-4, stage III-IV (*p* < 0.05) (Fig. 3f).

### Construction of a prognostic signature based on the TCGA cohort

**Fig. 4:**
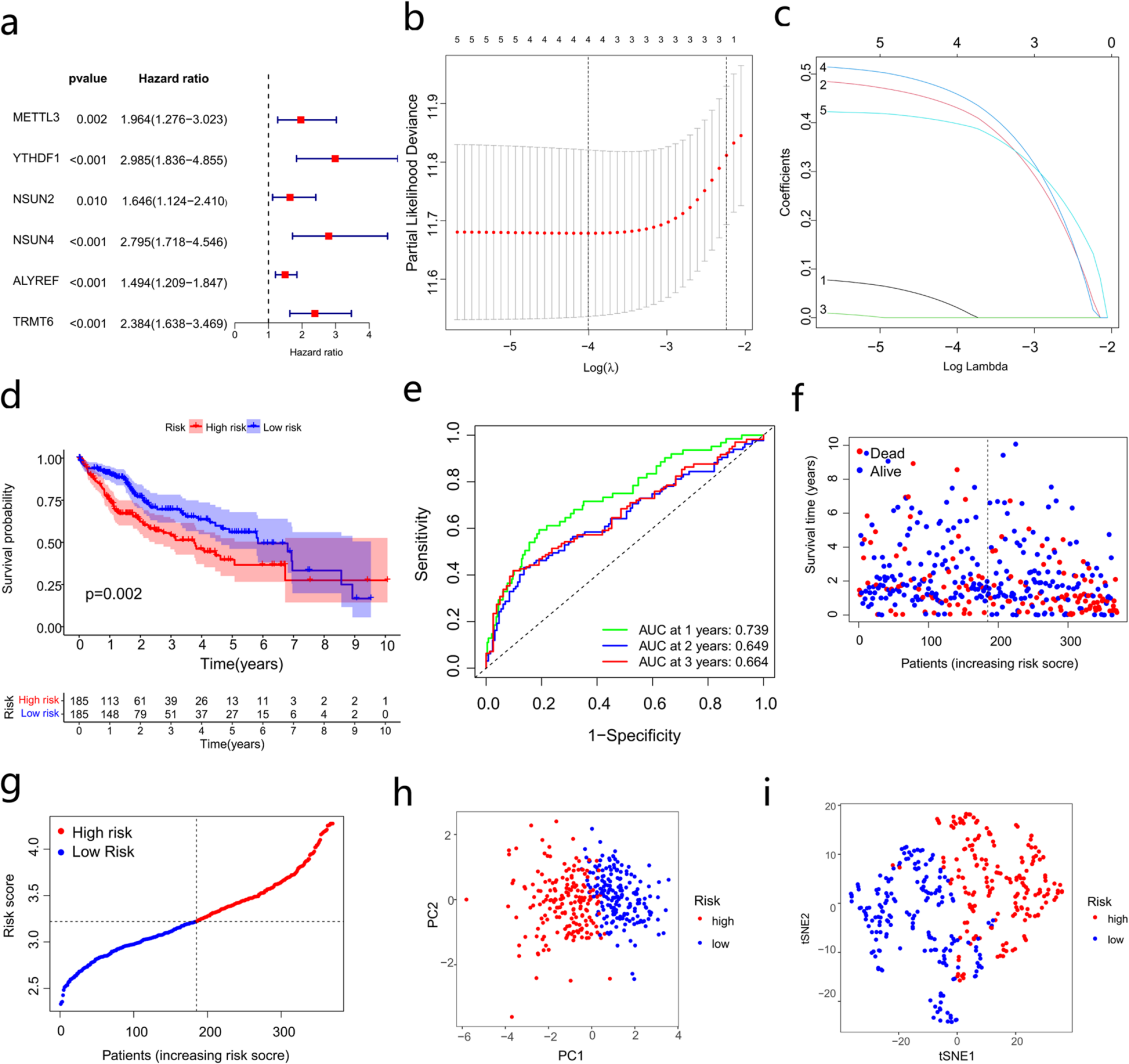
Establishing a prognostic signature based on m6A/m5C/m1A-related genes. **a** Univariate Cox regression analysis of m6A/m5C/m1A-related genes. **b**-**c** LASSO regression analysis and partial likelihood deviance of the prognostic genes. **d** OS analysis of different risk groups. **e** ROC curves about the prognostic signature predictive power. **f** Distribution of survival status in high- and low- risk groups. **g** Risk score curve about the high- and low-risk groups. **h** PCA and (**i**) tSNE results of the prognostic signature. LASSO, least absolute shrinkage and selection operator; OS, overall survival; ROC, receiver operating characteristic; PCA, principal component analysis; tSNE, t-distributed stochastic neighbor embedding

We used univariate Cox analysis to screen out prognostic m6A/m5C/m1A-related genes for subsequent analysis. A total of 6 genes were identified (Fig. 4a). Furthermore, we used the TCGA dataset as the training set (*n* = 370). LASSO regression analysis was performed on 6 prognostic m6A/m5C/m1A-related genes to further select the best prognostic indicators (Fig. 4b-c). Finally, 4 prognostic genes (METTL3, YTHDF1, NSUN4, and TRMT6) were retained with the least partial likelihood deviation. We developed a risk score model based on the following formula: risk score = (0.02* expression of METTL3) + (0.43* expression of YTHDF1) + (0.46* expression of NSUN4) + (0.40* expression of TRMT6). A total of 370 HCC patients in the training set were divided into high- and low-risk groups based on the median risk score, and the difference in OS between the high- and low-risk groups was statistically significant (*p* = 0.002, Fig. 4d). The areas under the ROC curve of the risk signature for the 1-, 2-, and 3-year periods were 0.739, 0.649, and 0.664, respectively (Fig. 4e). Compared with patients in the low-risk group, patients in the high-risk group showed shorter survival time and higher risk of death (Fig. 4f-g). PCA and t-SNE (Fig. 4h-i) showed that the patients were well divided into two risk groups.

### Validation of the prognostic signature in an external cohort

**Fig. 5:**
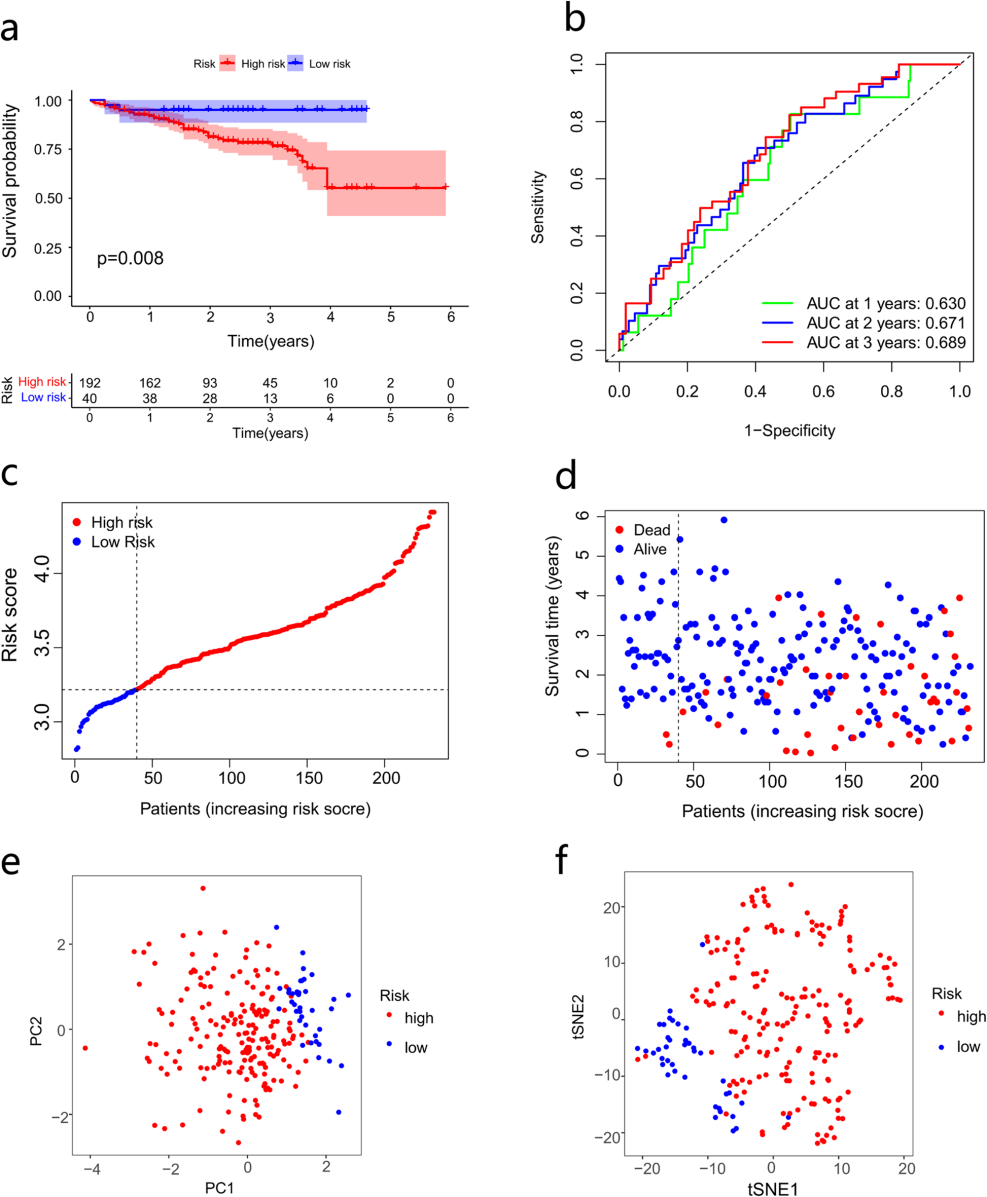
Validation of the prognostic signature based on the ICGC cohort. **a** OS analysis of risk groups. **b** ROC curves about the signature predictive power. **c** Risk score curve about the high- and low-risk groups. **d** Distribution of patients’ survival status. **e** PCA and (**f**) tSNE results of risk groups. ICGC, international cancer genome consortium; OS, overall survival; ROC, receiver operating characteristic; PCA, principal component analysis; tSNE, t-distributed stochastic neighbor embedding

A total of 232 HCC patients in the ICGC database were used as the external validation cohort. According to the median risk score in the training set, 192 patients in the ICGC cohort were assigned to the high-risk group, and 40 patients were assigned to the low-risk group. There was a significant difference in OS between the high- and low-risk groups (*p* = 0.008, Fig. 5a). Time-dependent ROC analysis was used to assess the sensitivity and specificity of the prognostic model, and areas under curves for 1-, 2- and 3-year survival were 0.630, 0.671, and 0.689, respectively (Fig. 5b). Compared with patients in the low-risk group, patients in the high-risk group had a higher risk of death and shorter survival time (Fig. 5c-d). PCA and t-SNE (Fig. 5e-f) showed that the patients were well divided into two risk groups.

### Independent prognostic value of the prognostic signature

**Fig. 6:**
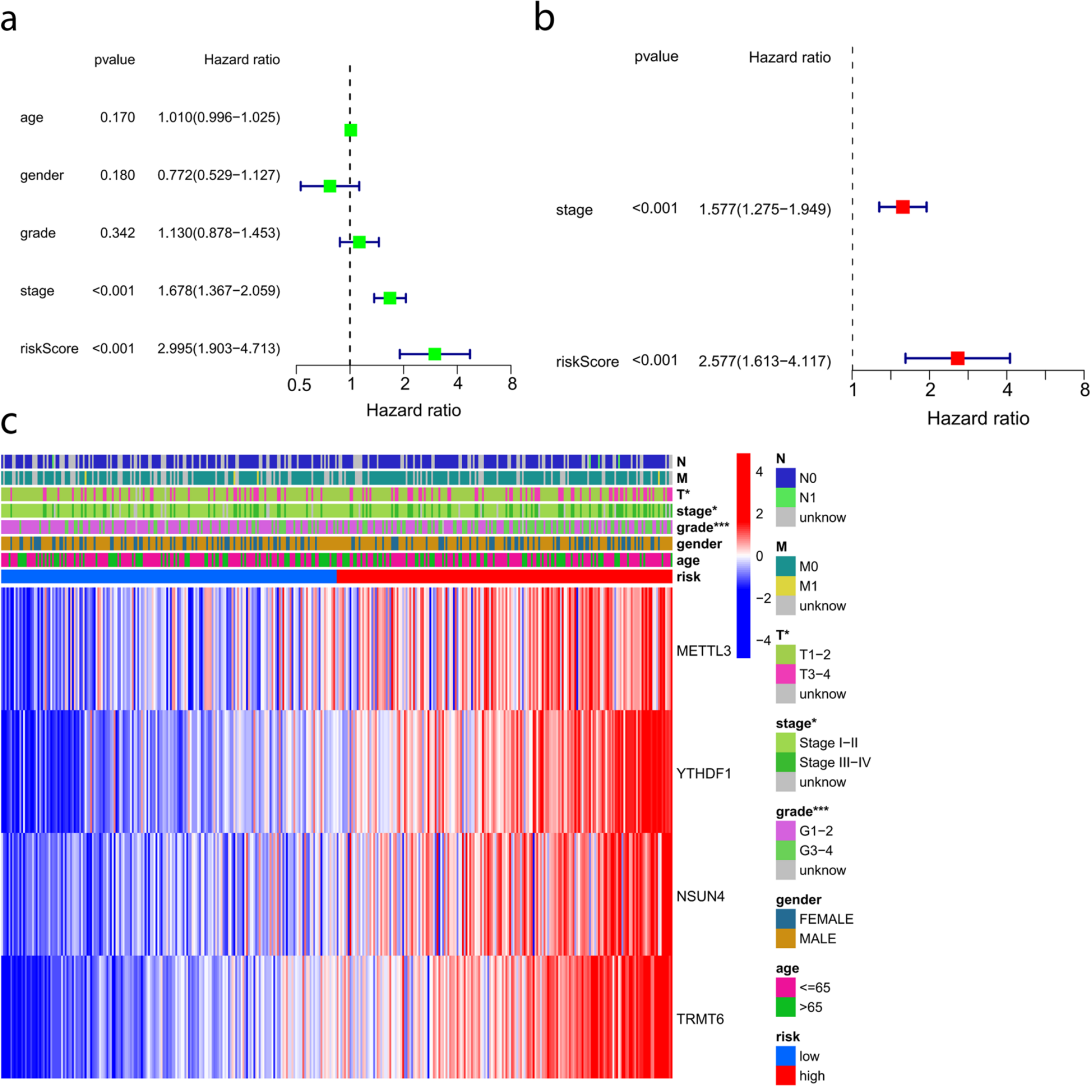
Correlation analysis between risk score and clinicopathological features. **a** Univariate and (**b**) multivariate Cox regression analyses. **c** Relationship between risk score and clinicopathological features

We used univariate and multivariate Cox regression analyses to assess whether the risk score of the m6A/m5A/m1A-related gene prognostic signature could serve as an independent prognostic factor. Univariate Cox regression analysis showed the risk score was an independent factor for predicting poorer survival (HR = 2.995, 95% CI: 1.903–4.713, Fig. 6a). Multivariate analysis showed that the risk score was a prognostic factor after adjustment for other confounding factors (HR = 2.577, 95% CI: 1.613–4.117, Fig. 6b). Additionally, we drew a heatmap to analyze the relationship between m6A/m5C/m1A-related genes and clinical characteristics in the TCGA cohort (Fig. 6c) and found that the risk score was correlated with grade and stage in HCC patients (*p* < 0.05).

### Validation of the expression of the 4 prognostic genes

**Fig. 7:**
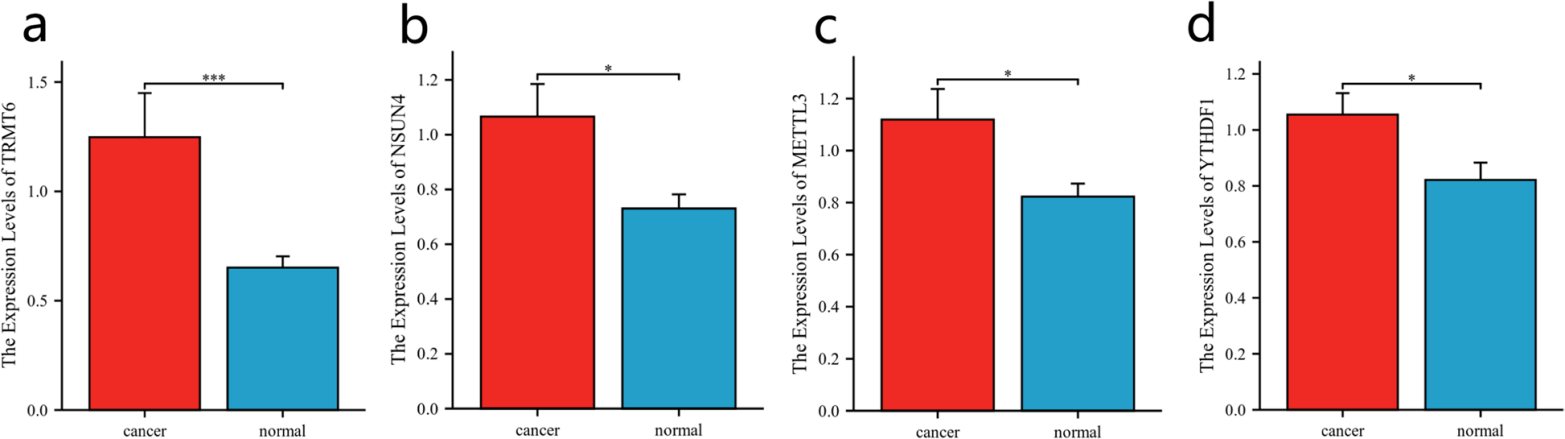
Validation of the expression of the prognostic signature’s genes. The expression of (**a**) TRMT6, (**b**) NSUN4, (**c**) METTL3 and (**d**) YTHDF in liver cancer and normal tissues. *p < 0.05, **p < 0.01, and ***p < 0.001

The expression levels of 4 prognostic genes were determined using RT‒qPCR in 30 HCC and 30 neighboring normal tissues. As shown in Fig. 7a-d, TRMT6, NSUN4, METTL3 and YTHDF1 were significantly overexpressed in HCC tissues (*p* < 0.05).

### Functional enrichment analysis based on the different risk groups

**Fig. 8:**
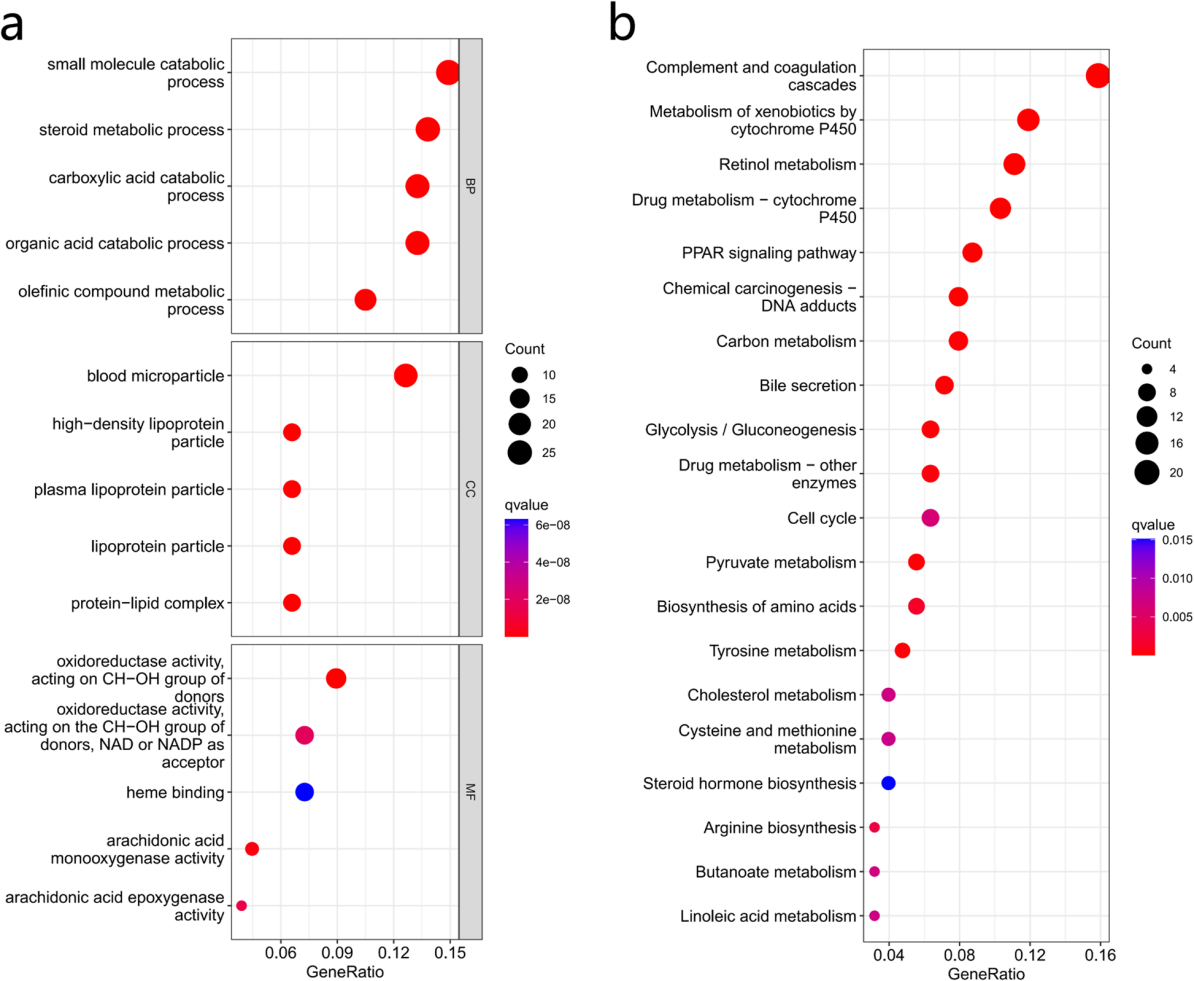
Functional enrichment analysis. **a** GO and (**b**) KEGG analysis about DEGs in the low- and high-risk groups. DEGs, differentially expressed genes; GO, Gene Ontology; KEGG, Kyoto Encyclopedia of Genomes

To further explore differences in gene function and pathways between risk groups, we employed the "limma" R package to extract DEGs with FDR < 0.05 and |log2FC|≥ 1. In the TCGA cohort, a total of 187 DEGs were identified between the low- and high-risk groups. Gene Ontology (GO) and Kyoto Encyclopedia of Genes and Genomes (KEGG) analyses were performed based on these DEGs. The results showed that DEGs were associated with small molecule catabolic process, steroid metabolic process, carboxylic acid catabolic process, organic acid catabolic process, olefinic compound metabolic process, retinol metabolism (Fig. 8a-b).

### Comparison of the immune microenvironment between high- and low-risk groups

**Fig. 9:**
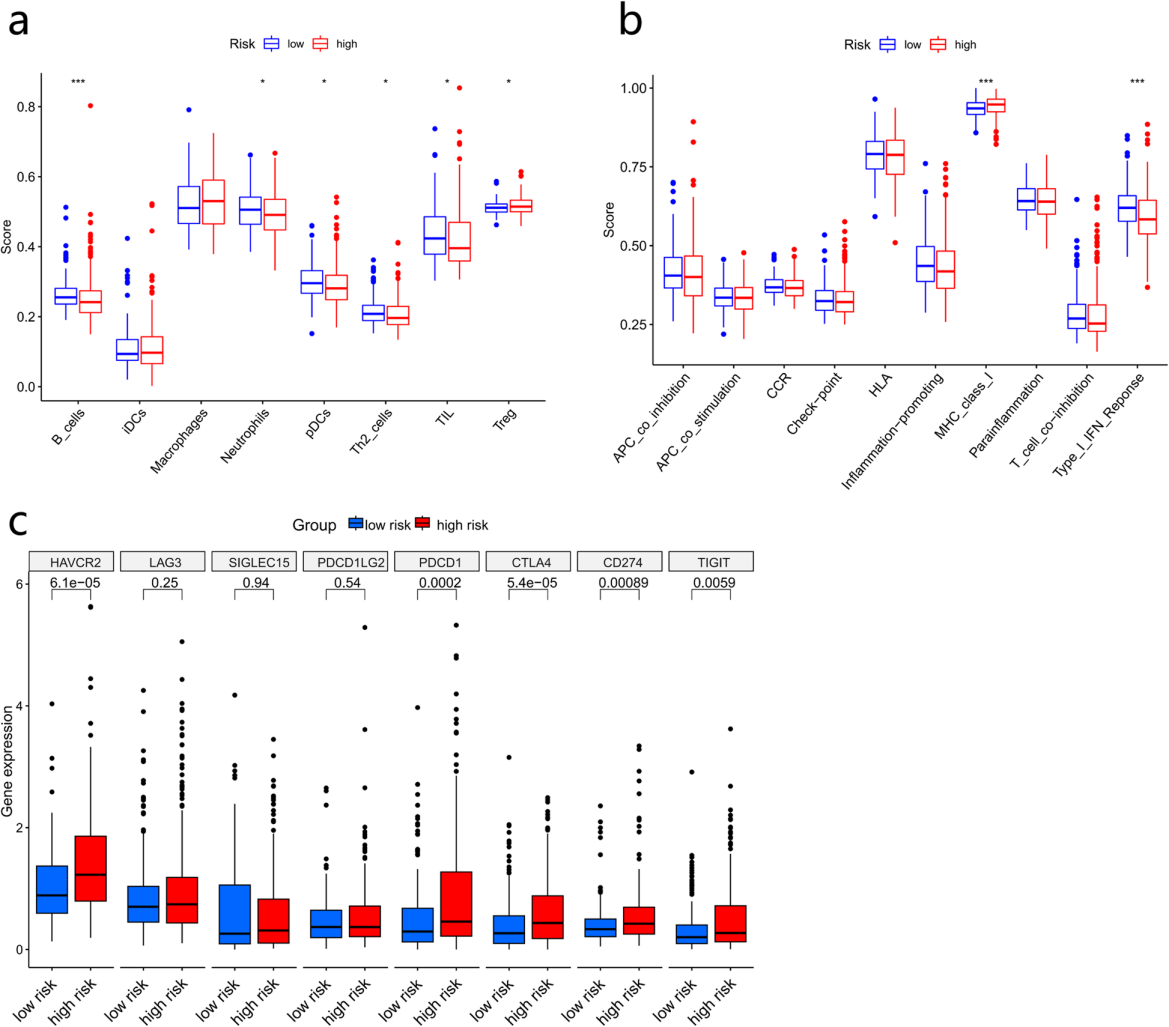
Correlation analysis between risk score and immune microenvironment. Boxplots show the scores of immune cells (**a**) and immune-related functions (**b**) in different risk groups. **c** Expression of immune checkpoints in differential risk groups. *, *P* < 0.05; **, *P* < 0.01; ***, *P* < 0.001

We used the ssGSEA method to compare the enrichment scores of 8 immune cell activities and 10 immune-related pathways between the high- and low-risk groups. The levels of B cells, neutrophils, DCs, Th2 cells and TIL expression levels were significantly increased in the low-risk group (Fig. 9a). The high-risk subgroup generally had lower activity for all immune pathways, except the MHC I pathway (Fig. 9b). High-risk group patients showed significant overexpression of HAVCR2, PDCD1, CTLA4, CD274, and TIGIT (*p* < 0.05) (Fig. 9c).

### Mutation analysis

**Fig. 10:**
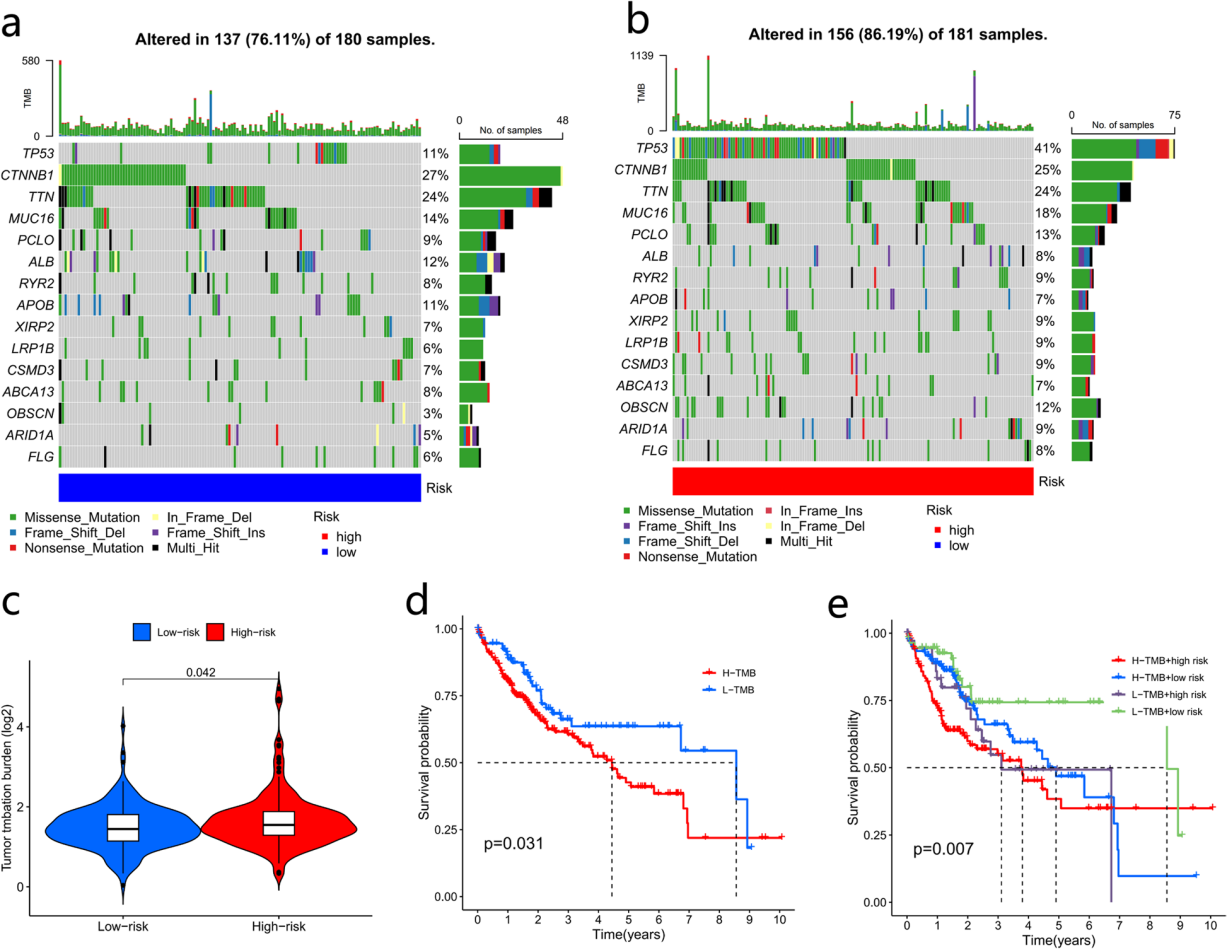
Gene mutation analysis based on the prognostic signature. Analysis of gene mutation frequencies in the low-risk (**a**) and high-risk (**b**) groups. **c** Analysis of TMB differences between the high-risk and low-risk groups. **d** Survival analysis based on tumor mutation burden. **e** Survival analysis based on TMB combined with the risk score. TMB, tumor mutation burden

Gene mutation analysis showed that the frequency of gene mutations was higher in the high-risk group than in the low-risk group (Fig. 10a-b). Patients in the high-risk group had increased TMB compared with those in the low-risk group (*p* < 0.05) (Fig. 10c). Patients in the high-TMB group had worse OS than those in the low-TMB group (*p* < 0.05) (Fig. 10d). The survival/prognosis of patients with high TMB in the high-risk group was significantly worse than that of patients with low TMB (Fig. 10e).

### Development of a nomogram to predict survival

**Fig. 11:**
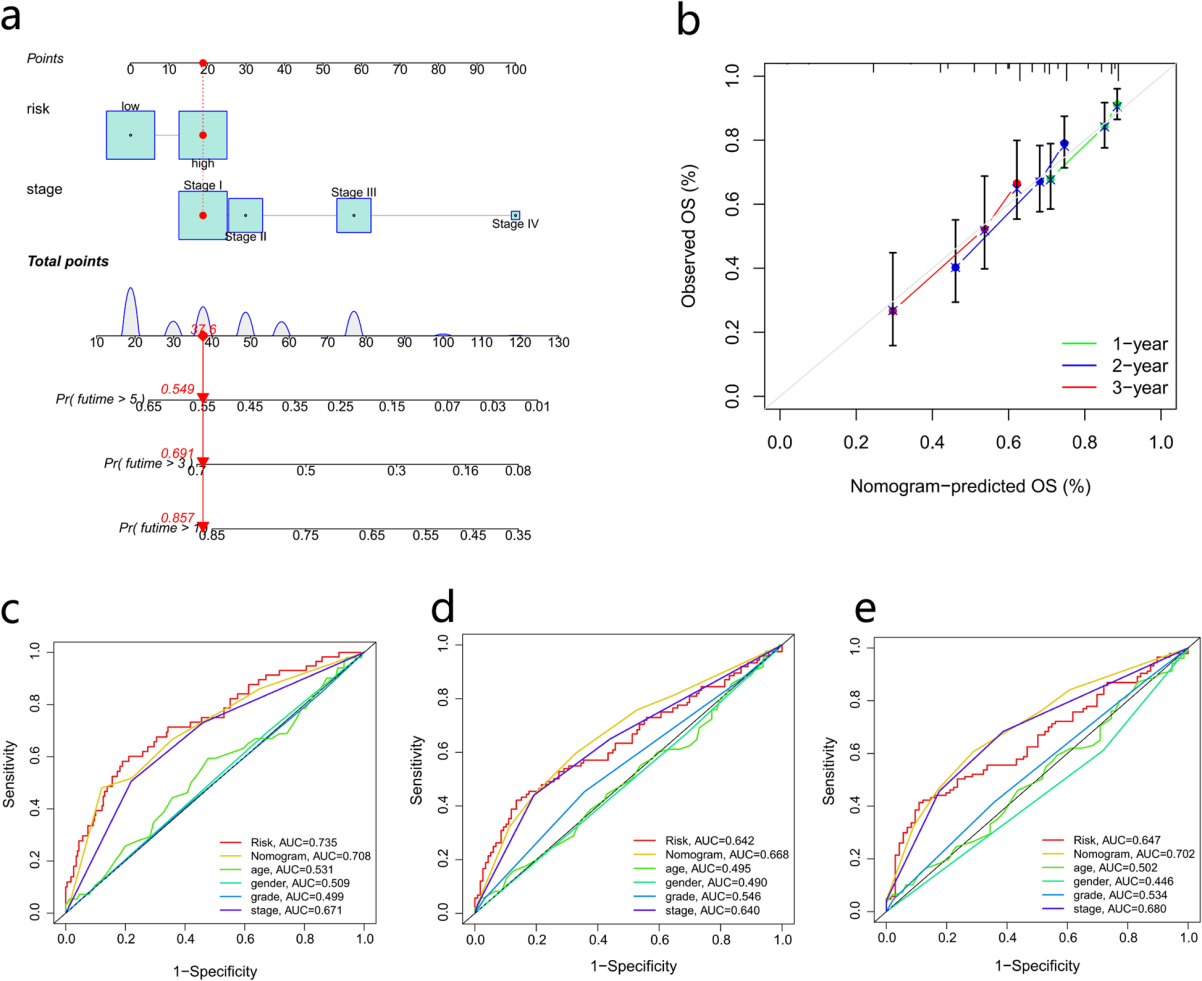
Establishment and validation of the nomogram based on clinicopathological characteristics and risk score. **a** Nomogram for risk score and clinical characteristics in patients with HCC. **b** Calibration analysis of the risk score containing nomogram for 1-, 2-, 3- year OS. **c** 1-year, (**d**) 2-year and (**e**) 3-year ROC analysis for the nomogram. *p < 0.05, **p < 0.01, and ***p < 0.001. HCC, hepatocellular carcinoma; OS, overall survival; ROC, receiver operating characteristic curve

We established a nomogram containing the risk score and clinicopathological characteristics to predict overall survival (Fig. 11a). Calibration curves of the nomogram for predicting 1-, 2- and 3-year OS suggested that the performance of the proposed nomogram was ideal (Fig. 11b). In predicting the survival/prognosis of patients, the 1-, 2- and 3-year ROC AUCs of the nomogram were better than those of clinicopathological features (Fig. 11c-e).

## Discussion

Nonmutational epigenetic reprogramming is considered to be an important cancer hallmark, and epigenetic modification is closely related to the occurrence and development of tumors [21]. Recent studies have demonstrated that RNA modifications may affect RNA metabolism, splicing, stability and translation, thereby affecting gene expression [22, 23]. RNA methylation is an important epigenetic modification that does not alter gene sequence but may play an essential role in multiple biological processes, such as gene expression, genome editing, and cell differentiation. With advances in RNA detection, various forms of RNA methylation, including m6A, m5C, m1A,, can be assessed.

A large number of studies have shown that RNA methylation modification was associated with the development of various malignant tumors. Cui et al. [24] found that m6A is involved in the regulation of glioma stem cell (GSC) self-renewal and differentiation. Knocking down the expression of the methyltransferase METTL3 and METTL14 proteins in cells can induce the mRNA expression of the proto-oncogenes ADAM19, EPHA3 and KLF4 in GSCs and promote the growth, self-renewal and differentiation of GSCs. Liu et al. [25] found that m6A methylation of EphA2 and VEGFA can promote angiogenesis in colorectal cancer. Wu et al. [26] found that protein arginine methyltransferase 5 (PRMT5) contributes to doxorubicin resistance in breast cancer by enhancing nuclear translocation of the RNA demethylase ALKBH5. In addition, a number of studies have confirmed that m6A modification is closely related to tumor immunotherapy [27–29], chemo-radiotherapy [30], and targeted therapy [31, 32] resistance. Hu et al. [33] found that m5C RNA methyltransferase NSUN2 involved in the proliferation, invasion and migration of gastric cancer cells. Wang et al. [34] found that overexpression of ALYREF can promote bladder cancer cell proliferation through PKM2-mediated glycolysis. The known demethylases for m1A modification include ALKBH3 and ALKBH1; methyltransferase enzymes include the TRMT family; and RNA-binding proteins include YTH family proteins [35, 36]. A large number of studies have shown that m1A also closely related to tumor proliferation, invasion, metabolism, and immune microenvironment. In most studies, m1A writers TRMT6 and eraser ALKBH3 are associated with poorer prognosis in multiple cancers [37–40] In this study, we comprehensively analyzed the relationship between RNA methylation types and HCC. In this study, two different molecular subtypes were identified based on the expression of m6A/m5C/m1A-related genes. The overall survival and prognosis of HCC patients in the two subtypes were significantly different. Also, the clinicopathologic features of different molecular subtypes were significantly different. The risk prognosis model based on m6A/m5C/m1A related genes showed that patients in the high-risk group had a significantly worse prognosis. Our results further confirmed that RNA modification is widely involved in the occurrence and development of liver cancer and affects the prognosis of patients.

Previous studies have shown that RNA methylation closely related to the tumor microenvironment. Researchers found that ALKBH5 promotes HCC growth, metastasis and macrophage recruitment through the ALKBH5/MAP3K8 axis [41]. Liu et al. [42] found that METTL3 mediated m6A methylation modification of circIGF2BP3 to suppress CD8 + T cell responses and promote immune escape in non-small cell lung cancer. Gao et al. [43]found that m1A modification related to the immune microenvironment of colon cancer through bioinformatics research. An important aspect of our study is that we explored the correlation between m6A/m5C/m1A-related genes and tumor immune microenvironment in HCC patients. Interestingly, we found that tumor-infiltrating lymphocyte (TIL) and B-cell infiltration levels were significantly increased in the low-risk group. Previous studies have confirmed that the presence of TILs and B cells is associated with improved survival in HCC patients [44, 45]. Furthermore, except for the MHC response pathway, the activation level of immune pathways in the high-risk group was lower than that in the lower-risk group. Based on these findings, the poor survival outcomes of HCC patients in the high-risk group may be due to reduced levels of antitumor immunity.

With the further study of tumor immunology and molecular biology, immunotherapy provides a new direction for the treatment of tumors. This immunotherapy includes immune checkpoint inhibitors (ICIs), therapeutic antibodies, and cell therapy. Research on ICIs for CTLA-4, PD-1, and PD-L1 is booming, and clinical studies have demonstrated safety and efficacy [46, 47]. We evaluated immune checkpoint-associated gene expression in the low- and high-risk groups. The expression of immune checkpoints was significantly increased in the high-risk group. These findings indicated that the high-risk group of HCC patients with suppressed immune function may benefit from immune checkpoint inhibitor therapy. Accumulating evidence suggests that tumor mutational burden (TMB) can serve as an important prognostic marker in relation to immunotherapy [48, 49]. We also compared differences in TMB and observed that subgroups with low risk scores tended to have lower TMB, with a significant survival benefit in patients with low TMB compared with those with high TMB. These results suggest that the m6A/m5C/m1A-related prognostic signature can be used for individualized treatment of HCC patients.

However, our study still has some limitations. First, prognostic markers were established and validated using retrospective data, and their clinical applicability needs to be validated with prospective data. Second, the potential biological functions and specific molecular mechanisms of these three m6A/m5C/m1A-related genes need to be further investigated. Third, the correlation between the risk score and tumor immunity was not experimentally demonstrated.

## Conclusions

In conclusion, our study further confirmed that m6A, m5C and m1A are closely related to HCC. Furthermore, in the TCGA and ICGC cohorts, the risk score generated by the risk model based on the 4 m6A/m5C/m1A-related genes was an independent risk factor for predicting the overall survival/prognosis of HCC patients. DEGs between the low-risk and high-risk groups were associated with tumor immunity. The combined analysis of RNA methylation-related genes in this study provides a new prognostic signature for HCC patients and provides an important basis for further research on the relationship between RNA methylation and immune function in HCC patients.

## Supplementary Information

**Additional file 1: Table S1.** Clinical data of TCGA hepatocellular cancer dataset. **Table S2.** Clinical data of ICGC hepatocellular cancer dataset. **Table S3.** The primers’ sequences for qRT PCR analysis.

## Data Availability

The datasets analyzed during the current study are available in The Cancer Genome Atlas (TCGA) repository (https://portal.gdc.cancer.gov/), the International Cancer Genome Consortium (ICGC) repository (https://dcc.icgc.org/) and the STRING website (https://string-db.org/).

## Abbreviations

m6A: N6-methyladenosine
m5C: 5-Methylcytosine
m1A: N1-methyladenosine
HCC: Hepatocellular cancer
TCGA: The Cancer Genome Atlas database
ICGC: International Cancer Genome Consortium
CNV: Copy number variation
PPI: Protein–protein interaction
CDF: Cumulative distribution function
OS: Overall survival
LASSO: Least absolute shrinkage and selection operator
ROC: Receiver operating characteristic
DEGs: Differentially expressed genes
GO: Gene Ontology
KEGG: Kyoto Encyclopedia of Genomes
PCA: Principal component analysis
tSNE: T-distributed stochastic neighbor embedding
TMB: Tumor mutation burden
