# Supplementary material for: Development and validation of a novel prognostic signature based on m6A/m5C/m1A-related genes in hepatocellular carcinoma
Source: BMC Med Genomics. 2023 Jul 31;16:177. doi: 10.1186/s12920-023-01611-x (PMC10391842; doi:10.1186/s12920-023-01611-x)
Supplement: Supplementary file 1 — Additional file 1: Table S1. Clinical data of TCGA hepatocellular cancer dataset. Table S2. Clinical data of ICGC hepatocellular cancer dataset. Table S3. The primers’ sequences for qRT PCR analysis. [file 12920_2023_1611_MOESM1_ESM.docx]

Table S1. Clinical data of TCGA hepatocellular cancer dataset.

| id | futime | fustat | age | gender | grade | stage | T | M | N |
| --- | --- | --- | --- | --- | --- | --- | --- | --- | --- |
| TCGA-DD-A1EA | 2415 | alive | 68 | MALE | G2 | Stage II | T2 | M0 | N0 |
| TCGA-KR-A7K0 | 65 | death | 65 | MALE | G1 | Stage I | T1 | M0 | N0 |
| TCGA-DD-A4NS | 2456 | death | 61 | FEMALE | G2 | Stage I | T1 | M0 | N0 |
| TCGA-CC-A5UC | 347 | death | 63 | MALE | G3 | Stage IIIA | T3 | M0 | N0 |
| TCGA-G3-AAV7 | 361 | alive | 38 | MALE | G2 | Stage II | T2 | M0 | N0 |
| TCGA-DD-AAED | 763 | alive | 51 | MALE | G3 | Stage I | T1 | M0 | N0 |
| TCGA-DD-AAE0 | 555 | alive | 45 | FEMALE | G4 | Stage IIIA | T3a | M0 | N0 |
| TCGA-CC-A3M9 | 300 | death | 45 | MALE | G3 | Stage IIIA | T3 | M0 | N0 |
| TCGA-DD-A3A4 | 612 | death | 37 | MALE | G3 | Stage IIIA | T3 | M0 | N0 |
| TCGA-DD-AADB | 1242 | alive | 51 | MALE | G4 | Stage I | T1 | M0 | N0 |
| TCGA-G3-A25V | 860 | alive | 68 | MALE | G2 | Stage I | T1 | M0 | N0 |
| TCGA-DD-AAE7 | 644 | alive | 72 | MALE | G2 | Stage I | T1 | M0 | N0 |
| TCGA-LG-A9QC | 425 | alive | 48 | MALE | G2 | Stage I | T1 | M0 | NX |
| TCGA-DD-AAEH | 784 | alive | 73 | MALE | G2 | Stage I | T1 | M0 | N0 |
| TCGA-DD-AAW1 | 1989 | alive | 55 | MALE | G2 | Stage IIIA | T3 | M0 | N0 |
| TCGA-2Y-A9H9 | 697 | alive | 70 | MALE | G2 | Stage I | T1 | MX | N0 |
| TCGA-5C-AAPD | 20 | alive | 61 | MALE | G1 | Stage II | T2 | M0 | N0 |
| TCGA-EP-A2KA | 627 | death | 52 | FEMALE | G3 | Stage IIIA | T3a | MX | NX |
| TCGA-DD-A73B | 283 | death | 72 | FEMALE | G2 | Stage I | T1 | M0 | N0 |
| TCGA-UB-A7MD | 52 | death | 67 | MALE | G3 | Stage I | T1 | MX | N0 |
| TCGA-DD-A113 | 2425 | alive | 55 | FEMALE | G3 | Stage II | T2 | M0 | N0 |
| TCGA-5C-A9VH | 322 | alive | 70 | MALE | G2 | Stage I | T1 | M0 | N0 |
| TCGA-ED-A5KG | 854 | alive | 60 | FEMALE | G2 | Stage II | T2 | M0 | N0 |
| TCGA-BC-A10U | 837 | death | 69 | MALE | G2 | unknow | T2 | MX | NX |
| TCGA-DD-A39V | 643 | death | 77 | MALE | G3 | Stage II | T2 | M0 | NX |
| TCGA-ED-A627 | 423 | alive | 74 | MALE | G2 | Stage I | T1 | M0 | NX |
| TCGA-G3-AAV2 | 372 | alive | 50 | MALE | G1 | Stage I | T1 | M0 | N0 |
| TCGA-ZS-A9CD | 1386 | death | 73 | MALE | G2 | Stage II | T2 | MX | NX |
| TCGA-GJ-A9DB | 67 | death | 68 | MALE | G2 | Stage I | T1 | MX | N0 |
| TCGA-G3-AAV4 | 27 | death | 83 | FEMALE | G1 | Stage I | T1 | M0 | N0 |
| TCGA-DD-AADP | 458 | alive | 45 | MALE | G3 | Stage I | T1 | M0 | N0 |
| TCGA-DD-A4NL | 1711 | alive | 46 | MALE | G1 | Stage I | T1 | M0 | N0 |
| TCGA-CC-5261 | 97 | death | 44 | MALE | G2 | Stage II | T2 | M0 | N0 |
| TCGA-UB-A7MC | 500 | alive | 59 | MALE | G3 | Stage IIIA | T3a | MX | N0 |
| TCGA-CC-A7IF | 649 | death | 59 | MALE | G1 | Stage IIIA | T3 | M0 | N0 |
| TCGA-4R-AA8I | 262 | death | 66 | MALE | G2 | Stage II | T2 | MX | NX |
| TCGA-CC-5259 | 250 | alive | 60 | FEMALE | G2 | Stage IIIC | T4 | M0 | N0 |
| TCGA-2Y-A9GW | 1271 | death | 64 | MALE | G2 | Stage I | T1 | MX | N0 |
| TCGA-BC-A10S | 1423 | death | 81 | MALE | G1 | unknow | T3 | MX | NX |
| TCGA-UB-A7ME | 486 | alive | 51 | MALE | G2 | Stage I | T1 | MX | NX |
| TCGA-ED-A97K | 6 | alive | 54 | MALE | G2 | Stage IIIA | T3a | M0 | N0 |
| TCGA-XR-A8TF | 693 | death | 74 | MALE | G1 | Stage I | T1 | MX | NX |
| TCGA-CC-A7II | 399 | alive | 54 | MALE | G3 | Stage IIIA | T3 | M0 | N0 |
| TCGA-DD-AADQ | 436 | alive | 59 | MALE | G3 | Stage II | T2 | M0 | N0 |
| TCGA-K7-A6G5 | 512 | alive | 66 | MALE | G2 | Stage I | T1 | MX | N0 |
| TCGA-CC-5264 | 102 | death | 71 | MALE | G2 | Stage IIIA | T3 | M0 | N0 |
| TCGA-DD-A39Y | 171 | death | 67 | MALE | G3 | Stage I | T1 | M0 | NX |
| TCGA-ED-A8O6 | 56 | death | 50 | FEMALE | G3 | Stage IIIA | T3a | M0 | N0 |
| TCGA-DD-A1E9 | 2759 | death | 70 | MALE | G2 | Stage I | T1 | M0 | N0 |
| TCGA-DD-A1EC | 602 | alive | 20 | FEMALE | G3 | Stage I | T1 | M0 | N0 |
| TCGA-DD-A115 | 2542 | death | 53 | MALE | G2 | Stage IIIA | T3 | M0 | N0 |
| TCGA-HP-A5MZ | 91 | death | 78 | MALE | G2 | Stage I | T1 | M0 | NX |
| TCGA-DD-A1EK | 558 | death | 64 | FEMALE | G2 | Stage IVB | T4 | M1 | N0 |
| TCGA-DD-AAE2 | 638 | alive | 51 | MALE | G3 | Stage I | T1 | M0 | N0 |
| TCGA-CC-A5UE | 272 | death | 48 | MALE | G2 | Stage IIIB | T4 | M0 | N0 |
| TCGA-NI-A8LF | 799 | alive | 74 | MALE | G3 | Stage I | T1 | MX | NX |
| TCGA-BW-A5NO | 20 | alive | 50 | MALE | G2 | Stage IIIA | T3a | MX | NX |
| TCGA-CC-A5UD | 304 | death | 45 | MALE | G2 | Stage IIIA | T3 | M0 | N0 |
| TCGA-DD-A1EF | 394 | death | 57 | FEMALE | G3 | Stage I | T1 | M0 | N0 |
| TCGA-GJ-A3OU | 879 | alive | 59 | MALE | G2 | Stage I | T1 | MX | NX |
| TCGA-DD-AAVU | 2202 | alive | 46 | MALE | G2 | Stage II | T2 | M0 | N0 |
| TCGA-G3-A6UC | 671 | alive | 65 | MALE | G2 | Stage IIIB | T3b | M0 | N0 |
| TCGA-CC-A9FU | 0 | alive | 52 | FEMALE | G2 | Stage IIIA | T3a | M0 | N0 |
| TCGA-DD-AAD3 | 1295 | alive | 43 | MALE | G2 | Stage I | T1 | M0 | N0 |
| TCGA-2Y-A9GU | 1939 | alive | 55 | FEMALE | G2 | Stage I | T1 | MX | NX |
| TCGA-MI-A75G | 698 | alive | 63 | MALE | G2 | Stage II | T2 | M0 | N0 |
| TCGA-FV-A4ZP | 2486 | death | 78 | MALE | G2 | Stage IIIA | T3 | M0 | NX |
| TCGA-PD-A5DF | 639 | death | 58 | FEMALE | G2 | Stage IIIB | T4 | M0 | N0 |
| TCGA-DD-A3A9 | 931 | death | 64 | FEMALE | G2 | Stage IVB | T4 | M1 | N0 |
| TCGA-DD-AACA | 2301 | alive | 65 | MALE | G3 | Stage I | T1 | M0 | N0 |
| TCGA-DD-AAW2 | 1855 | alive | 69 | MALE | G2 | Stage I | T1 | M0 | N0 |
| TCGA-DD-AADD | 1231 | alive | 51 | MALE | G4 | Stage I | T1 | M0 | N0 |
| TCGA-XR-A8TG | 898 | alive | 58 | MALE | G2 | Stage I | T1 | M0 | NX |
| TCGA-UB-AA0V | 314 | alive | 69 | FEMALE | G1 | Stage I | unknow | MX | NX |
| TCGA-CC-A7IE | 217 | death | 57 | MALE | G2 | Stage IIIA | T3 | M0 | N0 |
| TCGA-BC-A69I | 387 | alive | 69 | MALE | G1 | Stage I | T1 | M0 | N0 |
| TCGA-DD-AAE3 | 566 | alive | 50 | MALE | G2 | Stage I | T1 | M0 | N0 |
| TCGA-CC-A1HT | 101 | death | 50 | MALE | G3 | Stage IIIA | T3 | M0 | N0 |
| TCGA-ZP-A9D1 | 21 | alive | 56 | FEMALE | G2 | unknow | T1 | MX | NX |
| TCGA-EP-A3JL | 303 | alive | 76 | MALE | G2 | Stage I | T1 | MX | NX |
| TCGA-CC-A7IL | 278 | death | 61 | MALE | G1 | Stage IIIA | T3 | M0 | N0 |
| TCGA-CC-5263 | 129 | death | 35 | MALE | G1 | Stage IIIA | T3 | M0 | N0 |
| TCGA-G3-A25X | 1779 | alive | 73 | MALE | G3 | Stage II | T2 | M0 | N0 |
| TCGA-WQ-A9G7 | 30 | alive | 71 | FEMALE | G3 | unknow | T3a | M0 | NX |
| TCGA-G3-A25Z | 655 | alive | 58 | MALE | G2 | Stage I | T1 | M0 | N0 |
| TCGA-DD-AACX | 170 | alive | 66 | MALE | G3 | Stage II | T2 | M0 | N0 |
| TCGA-DD-AAE6 | 141 | alive | 59 | FEMALE | G2 | Stage I | T1 | M0 | N0 |
| TCGA-UB-AA0U | 327 | alive | 60 | MALE | G2 | Stage II | T2 | MX | NX |
| TCGA-RG-A7D4 | 1098 | alive | 69 | MALE | G2 | Stage II | T2 | M0 | N0 |
| TCGA-MI-A75I | 630 | alive | 61 | MALE | G1 | unknow | T2 | MX | NX |
| TCGA-DD-AAVX | 1718 | alive | 38 | MALE | G2 | Stage II | T2 | M0 | N0 |
| TCGA-EP-A2KC | 19 | death | 62 | MALE | G3 | Stage I | T1 | MX | NX |
| TCGA-DD-AADN | 898 | alive | 59 | MALE | G4 | Stage I | T1 | MX | NX |
| TCGA-DD-A4NV | 2398 | alive | 61 | MALE | G1 | Stage IIIA | T3 | M0 | N0 |
| TCGA-T1-A6J8 | 23 | alive | 68 | MALE | G2 | unknow | T1 | M0 | NX |
| TCGA-DD-A4NK | 1210 | death | 80 | FEMALE | G2 | Stage IIIA | T3 | M0 | N0 |
| TCGA-CC-A7IJ | 382 | alive | 56 | MALE | G3 | Stage II | T2 | M0 | N0 |
| TCGA-DD-AADR | 2028 | alive | 58 | MALE | G3 | Stage I | T1 | M0 | N0 |
| TCGA-DD-A4NH | 917 | alive | 65 | FEMALE | G3 | Stage IIIB | T3b | M0 | N0 |
| TCGA-BD-A2L6 | 1363 | alive | 69 | MALE | G2 | unknow | T2 | MX | NX |
| TCGA-G3-A3CG | 673 | alive | 80 | MALE | G2 | Stage I | T1 | M0 | N0 |
| TCGA-RC-A6M3 | 0 | alive | 24 | MALE | G3 | Stage II | T2 | M0 | N0 |
| TCGA-DD-AACB | 2324 | alive | 74 | FEMALE | G3 | Stage I | T1 | M0 | N0 |
| TCGA-BC-A10Y | 711 | death | 76 | MALE | G3 | unknow | T4 | MX | NX |
| TCGA-CC-A9FW | 248 | alive | 68 | MALE | G2 | Stage IIIA | T3 | M0 | N0 |
| TCGA-DD-A1EL | 415 | death | 23 | MALE | G3 | Stage II | T2 | M0 | N0 |
| TCGA-DD-AAEE | 810 | alive | 55 | MALE | G4 | Stage I | T1 | M0 | N0 |
| TCGA-BC-A10W | 91 | death | 50 | MALE | G3 | unknow | T4 | MX | NX |
| TCGA-CC-A8HU | 344 | death | 39 | FEMALE | G3 | Stage IIIA | T3 | M0 | N0 |
| TCGA-RC-A7SK | 472 | alive | 59 | MALE | G3 | Stage I | T1 | M0 | N0 |
| TCGA-CC-5258 | 129 | death | 48 | MALE | G2 | Stage II | T2 | M0 | N0 |
| TCGA-UB-A7MF | 214 | death | 56 | MALE | G2 | Stage IIIA | T3a | MX | NX |
| TCGA-DD-AACL | 107 | death | 66 | FEMALE | G3 | Stage I | T1 | M0 | N0 |
| TCGA-BC-A10T | 837 | death | 76 | MALE | G1 | unknow | T4 | MX | NX |
| TCGA-DD-AACE | 2184 | alive | 62 | MALE | G3 | Stage I | T1 | M0 | N0 |
| TCGA-BC-A110 | 2116 | death | 51 | FEMALE | G1 | unknow | T1 | MX | NX |
| TCGA-ED-A7PY | 390 | alive | 20 | FEMALE | G3 | Stage II | T2 | M0 | NX |
| TCGA-DD-A4NR | 9 | death | 85 | FEMALE | G3 | Stage I | T1 | M0 | N0 |
| TCGA-BC-A8YO | 562 | alive | 66 | FEMALE | G3 | Stage IIIC | T4 | M0 | N0 |
| TCGA-DD-AADU | 554 | alive | 60 | MALE | G3 | Stage II | T2 | M0 | N0 |
| TCGA-BC-A10Z | 34 | death | 62 | FEMALE | G2 | Stage I | T1 | MX | N0 |
| TCGA-DD-AACK | 9 | alive | 70 | MALE | G2 | Stage I | T1 | M0 | N0 |
| TCGA-DD-AAEK | 1067 | alive | 51 | MALE | G3 | Stage II | T2 | M0 | N0 |
| TCGA-DD-AAW3 | 1633 | alive | 69 | MALE | G2 | Stage I | T1 | M0 | N0 |
| TCGA-2Y-A9GZ | 848 | death | 82 | FEMALE | G2 | Stage II | T2 | MX | NX |
| TCGA-DD-A73F | 1085 | alive | 77 | FEMALE | G1 | Stage I | T1 | M0 | N0 |
| TCGA-DD-AACS | 1804 | alive | 39 | MALE | G3 | Stage I | T1 | M0 | N0 |
| TCGA-ED-A66Y | 296 | death | 51 | FEMALE | G3 | Stage IIIA | T3a | M0 | N0 |
| TCGA-G3-A3CJ | 594 | alive | 52 | MALE | G2 | Stage II | T2 | M0 | N0 |
| TCGA-2Y-A9H2 | 1731 | alive | 64 | FEMALE | G3 | Stage I | T1 | MX | N0 |
| TCGA-ED-A7PZ | 6 | alive | 61 | MALE | G2 | Stage II | T2 | M0 | NX |
| TCGA-DD-AACI | 1618 | alive | 69 | MALE | G3 | Stage II | T2 | M0 | N0 |
| TCGA-DD-A116 | 1622 | death | 68 | MALE | G3 | Stage IIIA | T3 | M0 | N0 |
| TCGA-DD-AAEG | 719 | alive | 59 | FEMALE | G3 | Stage I | T1 | M0 | N0 |
| TCGA-DD-A114 | 1149 | death | 42 | MALE | G3 | Stage II | T2 | M0 | unknow |
| TCGA-ZS-A9CF | 2412 | alive | 64 | MALE | G2 | Stage II | T2 | MX | NX |
| TCGA-RC-A6M6 | 9 | alive | 75 | MALE | G3 | Stage II | T2 | M0 | NX |
| TCGA-5R-AA1C | 520 | alive | 57 | MALE | G2 | Stage II | T2 | M0 | N0 |
| TCGA-CC-5262 | 103 | death | 67 | MALE | G1 | Stage IIIC | T4 | M0 | N0 |
| TCGA-DD-AACO | 1876 | alive | 40 | MALE | G3 | Stage I | T1 | M0 | N0 |
| TCGA-ZP-A9CY | 782 | alive | 66 | FEMALE | G1 | unknow | T1 | MX | NX |
| TCGA-ZP-A9D4 | 395 | alive | 64 | FEMALE | G1 | unknow | T1 | MX | NX |
| TCGA-ZS-A9CE | 1241 | alive | 79 | FEMALE | G1 | Stage II | T2 | MX | NX |
| TCGA-DD-AAVS | 1823 | alive | 56 | MALE | G2 | Stage I | T1 | M0 | N0 |
| TCGA-XR-A8TC | 1339 | alive | 43 | FEMALE | G2 | Stage I | T1 | MX | NX |
| TCGA-DD-A39W | 827 | death | 29 | FEMALE | G2 | Stage III | T3 | M0 | N0 |
| TCGA-BC-A69H | 444 | alive | 64 | MALE | G3 | Stage II | T2 | M0 | NX |
| TCGA-DD-A4NA | 1008 | alive | 67 | FEMALE | G3 | Stage IIIC | T2 | M0 | N1 |
| TCGA-MI-A75E | 507 | alive | 61 | MALE | G2 | Stage IIIC | T4 | M0 | N0 |
| TCGA-DD-AAVZ | 1900 | alive | 38 | MALE | G2 | Stage I | T1 | M0 | N0 |
| TCGA-G3-A5SL | 621 | alive | 70 | MALE | G2 | Stage II | T2 | M0 | NX |
| TCGA-ED-A7XP | 400 | alive | 53 | FEMALE | G3 | Stage II | T2 | M0 | N0 |
| TCGA-EP-A2KB | 596 | death | 46 | FEMALE | G2 | Stage I | T1 | MX | NX |
| TCGA-DD-A4NI | 816 | alive | 67 | MALE | G2 | Stage II | T2 | M0 | NX |
| TCGA-CC-A9FV | 0 | alive | 57 | MALE | G2 | Stage IIIA | T3 | M0 | N0 |
| TCGA-WQ-AB4B | 395 | alive | 62 | MALE | G2 | Stage II | T2 | M0 | NX |
| TCGA-G3-AAV1 | 359 | death | 51 | MALE | G3 | Stage IIIC | T4 | M0 | N0 |
| TCGA-CC-A3MC | 363 | alive | 54 | MALE | G2 | Stage IIIA | T3 | M0 | N0 |
| TCGA-G3-AAUZ | 480 | alive | 48 | MALE | G2 | Stage I | T1 | M0 | N0 |
| TCGA-DD-AACM | 1769 | alive | 48 | MALE | G3 | Stage II | T2 | M0 | N0 |
| TCGA-DD-A11D | 1560 | death | 57 | FEMALE | G2 | Stage I | T1 | M0 | N0 |
| TCGA-DD-AACF | 365 | death | 68 | MALE | G3 | Stage I | T1 | M0 | N0 |
| TCGA-BD-A3EP | 409 | alive | 75 | FEMALE | G2 | Stage I | T1 | M0 | N0 |
| TCGA-ED-A4XI | 819 | alive | 58 | MALE | G3 | Stage II | T2 | M0 | N0 |
| TCGA-DD-A11A | 79 | alive | 67 | MALE | G3 | Stage I | T1 | M0 | N0 |
| TCGA-QA-A7B7 | 94 | alive | 48 | MALE | G2 | Stage II | T2 | MX | NX |
| TCGA-DD-AADL | 636 | alive | 58 | MALE | G4 | Stage I | T1 | M0 | N0 |
| TCGA-2Y-A9H6 | 357 | alive | 68 | FEMALE | G2 | Stage I | T1 | MX | NX |
| TCGA-2Y-A9GY | 757 | death | 64 | FEMALE | G3 | Stage II | T2 | MX | NX |
| TCGA-DD-A73E | 44 | alive | 66 | MALE | G1 | Stage I | T1 | M0 | N0 |
| TCGA-RC-A7SF | 579 | alive | 66 | MALE | G2 | Stage I | T1 | M0 | N0 |
| TCGA-BC-A3KG | 680 | alive | 68 | FEMALE | G3 | Stage II | T2 | M0 | N0 |
| TCGA-DD-AAVP | 2752 | alive | 48 | MALE | G1 | Stage I | T1 | M0 | N0 |
| TCGA-ED-A459 | 910 | alive | 47 | MALE | G2 | Stage II | T2 | M0 | N0 |
| TCGA-DD-AACW | 1424 | alive | 43 | MALE | G3 | Stage I | T1 | M0 | N0 |
| TCGA-3K-AAZ8 | 396 | alive | 65 | MALE | G1 | Stage IIIB | T3b | MX | NX |
| TCGA-G3-A7M5 | 447 | alive | 76 | MALE | G2 | Stage I | T1 | MX | NX |
| TCGA-FV-A3R2 | 194 | death | 75 | MALE | unknow | Stage I | T1 | MX | NX |
| TCGA-BD-A3ER | 1115 | alive | 62 | MALE | G2 | Stage II | T2 | MX | NX |
| TCGA-DD-AADY | 555 | alive | 55 | FEMALE | G2 | Stage I | T1 | M0 | N0 |
| TCGA-DD-AACY | 1450 | alive | 61 | MALE | G3 | Stage I | T1 | M0 | N0 |
| TCGA-DD-A1EI | 183 | alive | 46 | MALE | G2 | Stage I | T1 | M0 | N0 |
| TCGA-2Y-A9HB | 260 | alive | 66 | MALE | G2 | Stage I | T1 | MX | NX |
| TCGA-CC-A123 | 219 | alive | 24 | FEMALE | G1 | Stage IIIA | T3 | M0 | N0 |
| TCGA-XR-A8TE | 925 | alive | 16 | MALE | G1 | Stage IIIA | T3 | MX | N0 |
| TCGA-CC-A8HT | 140 | death | 74 | MALE | G2 | Stage IIIA | T3 | M0 | N0 |
| TCGA-G3-A5SM | 520 | alive | 58 | MALE | G3 | Stage II | T2 | M0 | NX |
| TCGA-ES-A2HS | 688 | death | 80 | MALE | G2 | Stage I | T1 | MX | NX |
| TCGA-FV-A2QQ | 729 | alive | 80 | MALE | G2 | Stage I | T1 | MX | N0 |
| TCGA-DD-AACU | 1567 | alive | 59 | MALE | G3 | Stage I | T1 | M0 | N0 |
| TCGA-RC-A7SH | 468 | alive | 42 | MALE | G3 | Stage II | T2 | M0 | N0 |
| TCGA-ZP-A9D2 | 765 | death | 51 | MALE | G2 | unknow | T2 | MX | NX |
| TCGA-DD-AACT | 1562 | alive | 69 | FEMALE | G2 | Stage I | T1 | M0 | N0 |
| TCGA-DD-AADG | 1145 | alive | 70 | MALE | G3 | Stage IIIA | T3a | M0 | N0 |
| TCGA-2Y-A9H0 | 3675 | alive | 49 | MALE | G1 | Stage IIIA | T3 | M0 | N0 |
| TCGA-G3-AAV6 | 65 | death | 53 | FEMALE | G3 | Stage IIIA | T3a | M0 | N0 |
| TCGA-DD-A73D | 693 | alive | 68 | FEMALE | G1 | Stage II | T2 | MX | NX |
| TCGA-FV-A2QR | 581 | death | 75 | MALE | G1 | Stage I | T1 | M0 | N0 |
| TCGA-DD-AADK | 1049 | alive | 68 | FEMALE | G3 | Stage II | T2 | M0 | N0 |
| TCGA-DD-A4NE | 660 | death | 75 | FEMALE | G3 | Stage IIIA | T3a | M0 | N0 |
| TCGA-CC-A7IG | 299 | death | 47 | MALE | G2 | Stage II | T2 | M0 | N0 |
| TCGA-DD-A1EB | 2017 | alive | 72 | FEMALE | G2 | Stage I | T1 | M0 | N0 |
| TCGA-DD-A118 | 3437 | alive | 77 | FEMALE | G2 | Stage II | T2 | M0 | N0 |
| TCGA-5R-AAAM | 46 | death | 65 | FEMALE | G2 | Stage II | T2 | M0 | N0 |
| TCGA-DD-AAVQ | 2728 | alive | 38 | MALE | G2 | Stage I | T1 | M0 | N0 |
| TCGA-G3-A25U | 1636 | alive | 63 | FEMALE | G3 | Stage I | T1 | M0 | N0 |
| TCGA-G3-A7M8 | 430 | alive | 31 | MALE | G1 | Stage I | T1 | MX | NX |
| TCGA-DD-A3A5 | 3125 | death | 66 | FEMALE | G2 | Stage III | T3 | M0 | N0 |
| TCGA-DD-AACZ | 171 | death | 63 | FEMALE | G4 | Stage I | T1 | M0 | N0 |
| TCGA-BC-A217 | 1397 | death | 75 | FEMALE | G3 | Stage II | T2 | M0 | NX |
| TCGA-ED-A7XO | 427 | alive | 29 | MALE | G2 | Stage IIIA | T3a | M0 | N0 |
| TCGA-FV-A495 | 1 | alive | 51 | FEMALE | G2 | Stage II | T2 | M0 | NX |
| TCGA-DD-A1EH | 1495 | alive | 23 | MALE | G3 | Stage III | T3 | M0 | N0 |
| TCGA-FV-A23B | 1852 | death | 70 | FEMALE | unknow | Stage II | T2 | M0 | N0 |
| TCGA-MI-A75C | 291 | alive | 64 | MALE | G3 | Stage I | T1 | M0 | N0 |
| TCGA-DD-A4NP | 3308 | alive | 32 | MALE | G3 | Stage I | T1 | M0 | N0 |
| TCGA-DD-AADF | 115 | death | 64 | FEMALE | G4 | Stage I | T1 | M0 | N0 |
| TCGA-DD-AAEA | 575 | alive | 65 | MALE | G3 | Stage I | T1 | M0 | N0 |
| TCGA-EP-A3RK | 363 | alive | 73 | MALE | G2 | Stage IIIA | T3a | MX | NX |
| TCGA-BC-4072 | 1490 | death | 74 | FEMALE | G3 | Stage IIIA | T3 | M0 | N0 |
| TCGA-FV-A3I0 | 848 | alive | 76 | FEMALE | G2 | Stage II | T2 | M0 | NX |
| TCGA-UB-A7MB | 601 | alive | 24 | MALE | G3 | Stage II | T2 | MX | NX |
| TCGA-ZP-A9CZ | 706 | alive | 72 | MALE | G1 | unknow | T1 | MX | NX |
| TCGA-MI-A75H | 747 | alive | 77 | MALE | unknow | unknow | unknow | MX | NX |
| TCGA-G3-A5SJ | 698 | alive | 59 | MALE | G2 | Stage I | T1 | M0 | NX |
| TCGA-DD-AAD0 | 137 | alive | 73 | FEMALE | G2 | Stage I | T1 | M0 | N0 |
| TCGA-ED-A82E | 408 | alive | 60 | FEMALE | G2 | Stage IIIA | T3a | M0 | N0 |
| TCGA-BC-A5W4 | 547 | death | 69 | MALE | G3 | Stage IIIA | T3a | M0 | NX |
| TCGA-DD-AAEI | 1531 | alive | 72 | MALE | G2 | Stage I | T1 | M0 | N0 |
| TCGA-2Y-A9GT | 1624 | death | 51 | MALE | G2 | Stage I | T1 | MX | NX |
| TCGA-XR-A8TD | 1030 | alive | 49 | FEMALE | G3 | Stage IIIB | T3 | M0 | N0 |
| TCGA-2Y-A9H4 | 1452 | alive | 68 | MALE | G2 | Stage I | T1 | MX | N0 |
| TCGA-DD-AACD | 381 | death | 48 | MALE | G4 | Stage I | T1 | M0 | N0 |
| TCGA-BC-A112 | 153 | death | 80 | MALE | G2 | unknow | T3 | MX | NX |
| TCGA-2Y-A9GV | 2532 | death | 54 | FEMALE | G1 | Stage I | T1 | MX | NX |
| TCGA-DD-AADJ | 1066 | alive | 70 | FEMALE | G3 | Stage I | T1 | M0 | N0 |
| TCGA-CC-5260 | 87 | death | 61 | FEMALE | G1 | Stage IIIC | T4 | M0 | N0 |
| TCGA-LG-A6GG | 387 | alive | 79 | FEMALE | G2 | Stage II | T2 | M0 | NX |
| TCGA-G3-AAV0 | 476 | alive | 58 | MALE | G2 | Stage I | T1 | M0 | N0 |
| TCGA-DD-AACQ | 432 | death | 50 | MALE | G3 | Stage II | T2 | M0 | N0 |
| TCGA-KR-A7K7 | 951 | alive | 61 | FEMALE | G1 | Stage II | T2 | M0 | N0 |
| TCGA-DD-A4NJ | 928 | alive | 54 | FEMALE | G2 | Stage II | T2 | M0 | N0 |
| TCGA-DD-AAVV | 2455 | alive | 56 | MALE | G3 | Stage II | T2 | M0 | N0 |
| TCGA-DD-AAC8 | 16 | death | 72 | MALE | G3 | Stage I | T1 | M0 | N0 |
| TCGA-G3-A25W | 935 | alive | 79 | FEMALE | G2 | Stage IIIB | T3b | M0 | N0 |
| TCGA-WJ-A86L | 345 | alive | 68 | FEMALE | G2 | Stage I | T1 | MX | NX |
| TCGA-CC-A3MB | 315 | death | 36 | MALE | G1 | Stage IIIA | T3 | M0 | N0 |
| TCGA-G3-A7M7 | 361 | alive | 65 | MALE | G1 | Stage I | T1 | MX | NX |
| TCGA-DD-AADC | 425 | death | 53 | MALE | G3 | Stage I | T1 | M0 | N0 |
| TCGA-DD-AACC | 1685 | death | 61 | MALE | G2 | Stage I | T1 | M0 | N0 |
| TCGA-G3-A25T | 1553 | alive | 45 | FEMALE | G2 | Stage IIIA | T3 | M0 | N0 |
| TCGA-G3-A3CH | 780 | alive | 53 | MALE | G2 | Stage IIIA | T3a | M0 | N0 |
| TCGA-HP-A5N0 | 752 | death | 90 | FEMALE | unknow | unknow | TX | M0 | NX |
| TCGA-DD-A3A7 | 419 | death | 67 | MALE | G3 | Stage IIIB | T3b | M0 | N0 |
| TCGA-BC-4073 | 849 | alive | 73 | MALE | G3 | Stage IIIA | T3 | MX | N0 |
| TCGA-2Y-A9HA | 36 | death | 70 | MALE | G2 | Stage II | T2 | MX | NX |
| TCGA-WX-AA44 | 615 | alive | 64 | FEMALE | G3 | Stage I | T1 | MX | NX |
| TCGA-DD-A39Z | 601 | death | 43 | FEMALE | G2 | Stage II | T2 | M0 | NX |
| TCGA-DD-AAD5 | 1345 | alive | 54 | MALE | G3 | Stage I | T1 | M0 | N0 |
| TCGA-2Y-A9H7 | 1168 | alive | 81 | FEMALE | G2 | Stage I | T1 | MX | N0 |
| TCGA-DD-A1EJ | 1005 | death | 71 | FEMALE | G2 | Stage IIIC | T1 | M0 | N1 |
| TCGA-DD-A4NG | 802 | death | 77 | MALE | G2 | Stage IIIA | T3a | M0 | NX |
| TCGA-CC-A9FS | 211 | alive | 55 | MALE | G2 | Stage II | T2 | M0 | N0 |
| TCGA-DD-A4NO | 2245 | alive | 65 | MALE | G1 | Stage I | T1 | M0 | N0 |
| TCGA-DD-AAVY | 1970 | alive | 56 | MALE | G2 | Stage IIIA | T3 | M0 | N0 |
| TCGA-G3-A25Y | 452 | death | 52 | FEMALE | G3 | Stage I | T1 | M0 | N0 |
| TCGA-BC-A3KF | 8 | alive | 66 | FEMALE | G2 | Stage I | T1 | M0 | NX |
| TCGA-DD-A119 | 223 | death | 40 | MALE | G3 | Stage IV | T3a | M1 | N0 |
| TCGA-DD-AAVR | 2513 | alive | 44 | MALE | G2 | Stage I | T1 | M0 | N0 |
| TCGA-UB-A7MA | 848 | alive | 62 | FEMALE | G2 | Stage II | T2b | M0 | N0 |
| TCGA-ZS-A9CG | 341 | alive | 55 | MALE | G2 | Stage II | T2 | MX | NX |
| TCGA-ES-A2HT | 438 | death | 54 | MALE | G2 | Stage I | T1 | MX | NX |
| TCGA-G3-A7M9 | 56 | death | 70 | MALE | G2 | Stage IIIB | T3b | MX | NX |
| TCGA-DD-AADI | 1085 | alive | 43 | FEMALE | G3 | Stage I | T1 | M0 | N0 |
| TCGA-CC-A7IK | 262 | death | 59 | MALE | G3 | Stage IIIA | T3 | M0 | N0 |
| TCGA-DD-AADW | 587 | alive | 48 | MALE | G3 | Stage I | T1 | M0 | N0 |
| TCGA-FV-A4ZQ | 12 | alive | 52 | MALE | G2 | Stage I | T1 | M0 | NX |
| TCGA-DD-AADM | 12 | death | 58 | MALE | G3 | Stage II | T2 | M0 | N0 |
| TCGA-DD-AAW0 | 2015 | alive | 54 | MALE | G2 | Stage I | T1 | M0 | N0 |
| TCGA-K7-AAU7 | 359 | alive | 61 | MALE | G2 | Stage II | T2a | MX | NX |
| TCGA-ED-A8O5 | 406 | alive | 59 | FEMALE | G3 | Stage IIIA | T3a | M0 | N0 |
| TCGA-DD-A73G | 3478 | alive | 73 | FEMALE | G3 | Stage I | T1 | M0 | N0 |
| TCGA-DD-A11B | 14 | death | 73 | MALE | G2 | Stage I | T1 | M0 | N0 |
| TCGA-WX-AA46 | 756 | alive | 61 | MALE | G1 | Stage II | T2 | MX | NX |
| TCGA-ED-A66X | 406 | alive | 35 | MALE | G3 | Stage IIIA | T3a | M0 | N0 |
| TCGA-BC-A10X | 770 | death | 52 | FEMALE | G2 | Stage IIIA | T3a | MX | N0 |
| TCGA-DD-AADS | 474 | alive | 63 | MALE | G2 | Stage I | T1 | M0 | N0 |
| TCGA-MR-A520 | 229 | alive | 58 | MALE | G1 | Stage I | T1 | MX | NX |
| TCGA-LG-A9QD | 366 | alive | 68 | MALE | G2 | Stage IIIA | T3a | M0 | N0 |
| TCGA-FV-A496 | 10 | alive | 84 | FEMALE | G2 | Stage I | T1 | M0 | NX |
| TCGA-5C-A9VG | 328 | alive | 58 | MALE | G2 | Stage II | T2 | M0 | N0 |
| TCGA-BC-A10R | 308 | death | 66 | FEMALE | G2 | unknow | T3 | MX | NX |
| TCGA-DD-AA3A | 410 | death | 81 | FEMALE | G4 | Stage I | T1 | MX | N0 |
| TCGA-DD-A73A | 728 | alive | 71 | MALE | G2 | Stage I | T1 | M0 | N0 |
| TCGA-BW-A5NQ | 0 | alive | 63 | MALE | G3 | Stage I | T1 | MX | NX |
| TCGA-DD-A4NB | 989 | alive | 25 | MALE | G2 | Stage I | T1 | M0 | N0 |
| TCGA-DD-AADO | 453 | alive | 55 | MALE | G3 | Stage I | T1 | M0 | N0 |
| TCGA-FV-A3R3 | 366 | death | 38 | FEMALE | G2 | Stage I | T1 | MX | NX |
| TCGA-RC-A7SB | 588 | alive | 53 | MALE | G2 | Stage II | T2 | M0 | N0 |
| TCGA-DD-AAD2 | 658 | alive | 66 | MALE | G2 | Stage I | T1 | M0 | N0 |
| TCGA-DD-A1EG | 1372 | death | 76 | MALE | G3 | Stage I | T1 | M0 | N0 |
| TCGA-G3-A3CI | 180 | alive | 71 | MALE | G2 | Stage I | T1 | M0 | N0 |
| TCGA-DD-AAD1 | 564 | alive | 51 | FEMALE | G4 | Stage I | T1 | M0 | N0 |
| TCGA-DD-A11C | 662 | alive | 69 | MALE | G3 | Stage I | T1 | M0 | N0 |
| TCGA-WX-AA47 | 556 | death | 33 | FEMALE | G2 | Stage IIIA | T3a | MX | NX |
| TCGA-NI-A4U2 | 1791 | death | 71 | MALE | G1 | Stage IIIA | T3 | MX | NX |
| TCGA-2Y-A9H3 | 1516 | alive | 45 | MALE | G1 | Stage II | T2 | MX | NX |
| TCGA-BC-A216 | 1351 | alive | 62 | FEMALE | G2 | Stage IIIA | T3 | M0 | NX |
| TCGA-2Y-A9H5 | 555 | death | 59 | FEMALE | G3 | Stage I | T1 | MX | N0 |
| TCGA-EP-A12J | 570 | alive | 62 | MALE | G1 | Stage I | T1 | MX | NX |
| TCGA-DD-AACP | 415 | alive | 64 | MALE | G3 | Stage I | T1 | M0 | N0 |
| TCGA-DD-A3A2 | 2131 | death | 76 | FEMALE | G1 | Stage I | T1 | M0 | N0 |
| TCGA-DD-A3A0 | 785 | death | 70 | MALE | G2 | Stage I | T1 | M0 | NX |
| TCGA-DD-A4ND | 2746 | alive | 56 | FEMALE | G3 | Stage I | T1 | M0 | N0 |
| TCGA-G3-AAV5 | 354 | alive | 67 | MALE | G2 | Stage II | T2 | M0 | N0 |
| TCGA-DD-AAEB | 478 | alive | 60 | MALE | G2 | Stage I | T1 | M0 | N0 |
| TCGA-2Y-A9H8 | 633 | death | 85 | FEMALE | G2 | unknow | T1 | MX | NX |
| TCGA-G3-A25S | 416 | death | 64 | MALE | G2 | Stage I | T1 | M0 | N0 |
| TCGA-RC-A6M5 | 15 | alive | 20 | FEMALE | G2 | Stage IVA | T1 | M0 | N1 |
| TCGA-DD-AAE4 | 608 | alive | 49 | FEMALE | G1 | Stage I | T1 | M0 | N0 |
| TCGA-DD-AACH | 195 | death | 69 | MALE | G3 | Stage II | T2 | M0 | N0 |
| TCGA-DD-AAD8 | 1219 | alive | 73 | FEMALE | G2 | Stage I | T1 | M0 | N0 |
| TCGA-CC-A7IH | 365 | alive | 58 | MALE | G1 | Stage IIIA | T3 | M0 | N0 |
| TCGA-DD-AAE9 | 722 | alive | 69 | MALE | G3 | Stage I | T1 | M0 | N0 |
| TCGA-DD-AADA | 1233 | alive | 66 | FEMALE | G3 | Stage I | T1 | M0 | N0 |
| TCGA-DD-A39X | 1694 | death | 78 | FEMALE | G2 | Stage I | T1 | M0 | NX |
| TCGA-DD-AADE | 1202 | alive | 50 | MALE | G4 | Stage I | T1 | M0 | N0 |
| TCGA-K7-A5RG | 519 | alive | 66 | MALE | G1 | Stage I | T1 | MX | NX |
| TCGA-DD-AAVW | 2317 | alive | 35 | MALE | G2 | Stage I | T1 | M0 | N0 |
| TCGA-G3-A5SI | 768 | death | 44 | MALE | G2 | Stage II | T2 | M0 | N0 |
| TCGA-MR-A8JO | 330 | alive | 34 | MALE | G3 | Stage I | T1 | MX | N0 |
| TCGA-DD-AAE1 | 552 | alive | 52 | MALE | G3 | Stage I | T1 | M0 | N0 |
| TCGA-DD-A4NN | 899 | death | 56 | FEMALE | G3 | Stage I | T1 | M0 | N0 |
| TCGA-BW-A5NP | 0 | alive | 26 | FEMALE | G3 | Stage IV | T2 | M1 | N0 |
| TCGA-CC-A8HV | 279 | death | 51 | FEMALE | G2 | Stage II | T2 | M0 | N0 |
| TCGA-G3-A3CK | 585 | alive | 61 | MALE | G2 | Stage I | T1 | M0 | N0 |
| TCGA-EP-A26S | 608 | alive | 70 | MALE | G2 | Stage I | T1 | MX | N0 |
| TCGA-2Y-A9GX | 2442 | alive | 68 | MALE | G2 | Stage I | T1 | MX | NX |
| TCGA-RC-A7S9 | 640 | alive | 47 | FEMALE | G3 | Stage I | T1 | M0 | N0 |
| TCGA-YA-A8S7 | 412 | death | 68 | MALE | G3 | Stage IIIA | T3a | MX | N0 |
| TCGA-DD-A3A3 | 535 | death | 45 | MALE | G2 | Stage I | T1 | M0 | N0 |
| TCGA-O8-A75V | 538 | alive | 54 | MALE | G2 | Stage I | T1 | MX | NX |
| TCGA-ZP-A9D0 | 1091 | alive | 67 | FEMALE | G1 | unknow | T1 | MX | NX |
| TCGA-DD-A1EE | 349 | death | 73 | MALE | G3 | Stage IIIA | T3 | M0 | N0 |
| TCGA-DD-A3A6 | 3258 | death | 72 | FEMALE | G2 | Stage II | T2 | M0 | N0 |
| TCGA-RC-A6M4 | 22 | alive | 74 | FEMALE | G2 | Stage IIIA | T3 | MX | NX |
| TCGA-DD-AAD6 | 672 | alive | 66 | MALE | G3 | Stage IIIA | T3a | M0 | N0 |
| TCGA-K7-A5RF | 631 | alive | 64 | MALE | G1 | Stage I | T1 | MX | NX |
| TCGA-CC-A8HS | 300 | death | 18 | MALE | G1 | Stage IIIC | T3 | M0 | N1 |
| TCGA-G3-A5SK | 744 | alive | 58 | MALE | G1 | Stage I | T1 | M0 | NX |
| TCGA-DD-A4NQ | 373 | death | 60 | MALE | G3 | Stage II | T2 | M0 | N0 |
| TCGA-DD-A1ED | 2301 | alive | 68 | MALE | G1 | Stage I | T1 | M0 | N0 |
| TCGA-G3-AAV3 | 412 | alive | 58 | FEMALE | G2 | Stage II | T2 | M0 | N0 |
| TCGA-ZP-A9CV | 1088 | death | 59 | MALE | G1 | unknow | T1 | MX | NX |
| TCGA-DD-AACV | 1531 | alive | 53 | MALE | G3 | Stage I | T1 | M0 | N0 |
| TCGA-DD-AACG | 469 | death | 52 | MALE | G4 | Stage II | T2 | M0 | N0 |
| TCGA-CC-A3MA | 303 | death | 61 | MALE | G2 | Stage IIIA | T3 | M0 | N0 |
| TCGA-2V-A95S |  | alive | unknow | MALE | G3 | Stage II | T2 | MX | NX |
| TCGA-2Y-A9H1 | 1229 | death | 58 | MALE | G2 | Stage I | T1 | MX | NX |
| TCGA-DD-A4NF | 942 | alive | 72 | MALE | G2 | Stage I | T1 | M0 | N0 |
| TCGA-DD-A3A1 | 233 | death | 65 | MALE | G2 | Stage IIIA | T3b | M0 | N0 |
| TCGA-KR-A7K2 | 829 | alive | 64 | MALE | G1 | Stage I | T1 | M0 | N0 |
| TCGA-DD-AAC9 | 347 | alive | 51 | MALE | G2 | Stage I | T1 | M0 | N0 |
| TCGA-DD-AACJ | 2102 | alive | 75 | MALE | G2 | Stage II | T2 | M0 | N0 |
| TCGA-DD-AACN | 1302 | alive | 32 | MALE | G3 | Stage I | T1 | M0 | N0 |
| TCGA-GJ-A6C0 | 31 | death | 75 | FEMALE | G2 | Stage II | T2 | MX | NX |
| TCGA-DD-AAE8 | 664 | alive | 45 | MALE | G3 | Stage I | T1 | M0 | N0 |
| TCGA-DD-AADV | 574 | alive | 50 | MALE | G3 | Stage I | T1 | M0 | N0 |
| TCGA-FV-A3I1 | 247 | death | 81 | FEMALE | G2 | Stage II | T2 | MX | N0 |
| TCGA-BC-A10Q | 1135 | death | 72 | FEMALE | unknow | unknow | T2 | MX | NX |
| TCGA-2Y-A9GS | 724 | death | 58 | MALE | G2 | unknow | T2 | MX | NX |
| TCGA-ED-A7PX | 6 | alive | 48 | FEMALE | G3 | Stage II | T2 | M0 | NX |
| TCGA-DD-A3A8 | 11 | death | 75 | MALE | G2 | Stage II | T2 | M0 | N0 |
| TCGA-G3-A7M6 | 632 | alive | 60 | FEMALE | G3 | Stage I | T1 | MX | NX |
| TCGA-KR-A7K8 | 906 | alive | 57 | MALE | G1 | Stage I | T1 | M0 | N0 |
| TCGA-5R-AA1D | 449 | alive | 17 | FEMALE | G3 | Stage IIIA | T3a | M0 | N0 |

Table S2. Clinical data of ICGC hepatocellular cancer dataset.

| id | futime | fustat | gender | age | stage |
| --- | --- | --- | --- | --- | --- |
| DO45275 | 10 | death | MALE | 65 | Stage III |
| DO23516 | 20 | death | MALE | 69 | Stage III |
| DO45309 | 30 | death | MALE | 73 | Stage III |
| DO50798 | 30 | death | MALE | 77 | Stage III |
| DO45145 | 60 | death | MALE | 78 | Stage III |
| DO45205 | 90 | death | FEMALE | 67 | Stage II |
| DO50832 | 90 | alive | MALE | 73 | Stage III |
| DO50785 | 90 | death | MALE | 37 | Stage IV |
| DO48687 | 120 | alive | FEMALE | 73 | Stage II |
| DO48682 | 120 | death | FEMALE | 72 | Stage III |
| DO45193 | 120 | death | FEMALE | 68 | Stage IV |
| DO48728 | 150 | alive | FEMALE | 74 | Stage II |
| DO50815 | 150 | alive | MALE | 62 | Stage III |
| DO23551 | 150 | death | MALE | 73 | Stage III |
| DO23511 | 180 | death | FEMALE | 83 | Stage III |
| DO50859 | 180 | alive | MALE | 77 | Stage II |
| DO23530 | 180 | death | MALE | 53 | Stage IV |
| DO48717 | 180 | death | MALE | 54 | Stage IV |
| DO45247 | 210 | alive | FEMALE | 31 | Stage II |
| DO50857 | 210 | alive | MALE | 72 | Stage II |
| DO50845 | 210 | alive | MALE | 72 | Stage III |
| DO45245 | 240 | death | FEMALE | 75 | Stage II |
| DO45307 | 240 | death | FEMALE | 54 | Stage IV |
| DO48741 | 240 | alive | MALE | 64 | Stage III |
| DO45211 | 240 | alive | MALE | 76 | Stage III |
| DO45217 | 270 | death | FEMALE | 69 | Stage III |
| DO45263 | 270 | death | MALE | 67 | Stage III |
| DO50842 | 300 | alive | FEMALE | 75 | Stage IV |
| DO45295 | 300 | alive | MALE | 76 | Stage II |
| DO45305 | 300 | alive | MALE | 66 | Stage IV |
| DO50844 | 300 | alive | MALE | 75 | Stage IV |
| DO50839 | 330 | alive | FEMALE | 47 | Stage III |
| DO50840 | 330 | alive | MALE | 49 | Stage II |
| DO45259 | 330 | alive | MALE | 65 | Stage III |
| DO45289 | 360 | alive | FEMALE | 78 | Stage III |
| DO45131 | 360 | death | MALE | 53 | Stage IV |
| DO23508 | 390 | alive | FEMALE | 79 | Stage II |
| DO48727 | 390 | death | FEMALE | 81 | Stage II |
| DO45221 | 390 | death | FEMALE | 75 | Stage IV |
| DO45195 | 390 | alive | MALE | 62 | Stage I |
| DO45287 | 390 | alive | MALE | 74 | Stage I |
| DO50816 | 390 | alive | MALE | 63 | Stage II |
| DO50825 | 390 | alive | MALE | 70 | Stage II |
| DO50817 | 390 | alive | MALE | 75 | Stage II |
| DO50813 | 420 | alive | MALE | 56 | Stage II |
| DO45127 | 420 | death | MALE | 73 | Stage III |
| DO50814 | 420 | alive | MALE | 83 | Stage III |
| DO50811 | 450 | alive | MALE | 62 | Stage I |
| DO23538 | 450 | alive | MALE | 67 | Stage II |
| DO48672 | 450 | alive | MALE | 81 | Stage II |
| DO45103 | 450 | alive | MALE | 47 | Stage III |
| DO45293 | 450 | alive | MALE | 68 | Stage III |
| DO50837 | 480 | alive | FEMALE | 53 | Stage I |
| DO50791 | 480 | death | FEMALE | 79 | Stage I |
| DO50827 | 480 | alive | FEMALE | 48 | Stage II |
| DO48757 | 480 | alive | FEMALE | 68 | Stage III |
| DO45273 | 480 | alive | MALE | 62 | Stage II |
| DO48751 | 480 | death | MALE | 68 | Stage II |
| DO45285 | 480 | alive | MALE | 59 | Stage III |
| DO50783 | 480 | alive | MALE | 62 | Stage III |
| DO50787 | 480 | alive | MALE | 63 | Stage III |
| DO50848 | 480 | alive | MALE | 74 | Stage III |
| DO50819 | 510 | alive | FEMALE | 72 | Stage II |
| DO50820 | 510 | alive | FEMALE | 77 | Stage III |
| DO50831 | 510 | alive | MALE | 52 | Stage II |
| DO50818 | 510 | alive | MALE | 65 | Stage II |
| DO50822 | 510 | alive | MALE | 66 | Stage II |
| DO50800 | 510 | alive | MALE | 74 | Stage II |
| DO48753 | 510 | alive | MALE | 55 | Stage III |
| DO45283 | 510 | alive | MALE | 60 | Stage III |
| DO45119 | 510 | death | MALE | 74 | Stage III |
| DO50803 | 540 | alive | FEMALE | 62 | Stage I |
| DO50799 | 540 | alive | FEMALE | 66 | Stage II |
| DO50807 | 540 | alive | MALE | 67 | Stage I |
| DO50796 | 540 | alive | MALE | 69 | Stage II |
| DO50834 | 540 | alive | MALE | 72 | Stage II |
| DO45161 | 540 | death | MALE | 74 | Stage II |
| DO50851 | 540 | alive | MALE | 80 | Stage II |
| DO48747 | 540 | alive | MALE | 62 | Stage III |
| DO50806 | 540 | alive | MALE | 70 | Stage IV |
| DO50793 | 540 | alive | MALE | 76 | Stage IV |
| DO48720 | 570 | alive | FEMALE | 62 | Stage II |
| DO48743 | 570 | alive | FEMALE | 69 | Stage II |
| DO45183 | 570 | death | FEMALE | 72 | Stage II |
| DO50789 | 570 | alive | FEMALE | 73 | Stage II |
| DO45123 | 570 | death | FEMALE | 78 | Stage II |
| DO50855 | 570 | alive | MALE | 47 | Stage II |
| DO23545 | 570 | death | MALE | 67 | Stage II |
| DO50808 | 570 | alive | MALE | 68 | Stage II |
| DO45291 | 570 | alive | MALE | 69 | Stage III |
| DO45267 | 570 | death | MALE | 73 | Stage III |
| DO48746 | 570 | alive | MALE | 76 | Stage III |
| DO50829 | 570 | alive | MALE | 78 | Stage III |
| DO48736 | 600 | alive | FEMALE | 76 | Stage I |
| DO45179 | 600 | alive | FEMALE | 78 | Stage II |
| DO48723 | 600 | alive | MALE | 73 | Stage I |
| DO48733 | 600 | alive | MALE | 72 | Stage II |
| DO48742 | 600 | alive | MALE | 76 | Stage II |
| DO45185 | 600 | alive | MALE | 89 | Stage II |
| DO45171 | 600 | alive | MALE | 56 | Stage III |
| DO45277 | 600 | alive | MALE | 64 | Stage III |
| DO48730 | 630 | alive | FEMALE | 73 | Stage II |
| DO45249 | 630 | alive | MALE | 57 | Stage I |
| DO23519 | 630 | alive | MALE | 65 | Stage III |
| DO48732 | 630 | alive | MALE | 73 | Stage III |
| DO50778 | 630 | alive | MALE | 60 | Stage IV |
| DO227801 | 660 | alive | FEMALE | 77 | Stage I |
| DO50772 | 660 | alive | MALE | 70 | Stage I |
| DO45133 | 660 | death | MALE | 64 | Stage III |
| DO50780 | 660 | death | MALE | 70 | Stage IV |
| DO48715 | 690 | death | FEMALE | 58 | Stage II |
| DO48760 | 690 | alive | FEMALE | 70 | Stage II |
| DO50776 | 690 | alive | MALE | 75 | Stage I |
| DO50805 | 690 | alive | MALE | 72 | Stage II |
| DO45165 | 690 | alive | MALE | 75 | Stage II |
| DO48719 | 690 | alive | MALE | 80 | Stage II |
| DO50774 | 720 | alive | FEMALE | 68 | Stage II |
| DO48737 | 720 | alive | MALE | 52 | Stage I |
| DO45147 | 720 | death | MALE | 57 | Stage II |
| DO48716 | 720 | alive | MALE | 71 | Stage II |
| DO45093 | 720 | death | MALE | 71 | Stage II |
| DO48695 | 720 | alive | MALE | 77 | Stage II |
| DO50804 | 720 | alive | MALE | 81 | Stage II |
| DO50809 | 720 | death | MALE | 56 | Stage III |
| DO48759 | 720 | alive | MALE | 73 | Stage III |
| DO50850 | 720 | alive | MALE | 74 | Stage III |
| DO45255 | 720 | alive | MALE | 81 | Stage III |
| DO48704 | 750 | alive | FEMALE | 68 | Stage III |
| DO23540 | 750 | alive | MALE | 73 | Stage I |
| DO48703 | 750 | alive | MALE | 64 | Stage II |
| DO45269 | 750 | alive | MALE | 59 | Stage III |
| DO45281 | 750 | alive | MALE | 83 | Stage IV |
| DO48725 | 780 | alive | MALE | 66 | Stage I |
| DO48701 | 780 | alive | MALE | 78 | Stage II |
| DO23509 | 780 | death | MALE | 60 | Stage III |
| DO48738 | 780 | alive | MALE | 83 | Stage IV |
| DO23552 | 810 | alive | FEMALE | 80 | Stage I |
| DO48697 | 810 | death | FEMALE | 64 | Stage II |
| DO48700 | 810 | alive | FEMALE | 78 | Stage II |
| DO48684 | 810 | death | FEMALE | 71 | Stage III |
| DO48712 | 810 | alive | MALE | 50 | Stage II |
| DO45257 | 810 | alive | MALE | 46 | Stage III |
| DO23548 | 840 | alive | FEMALE | 78 | Stage I |
| DO23534 | 840 | alive | MALE | 60 | Stage II |
| DO48694 | 840 | alive | MALE | 71 | Stage II |
| DO45115 | 840 | alive | MALE | 81 | Stage II |
| DO23510 | 870 | alive | FEMALE | 80 | Stage II |
| DO45239 | 870 | alive | MALE | 42 | Stage II |
| DO45253 | 870 | alive | MALE | 50 | Stage II |
| DO48761 | 870 | alive | MALE | 55 | Stage II |
| DO48706 | 870 | alive | MALE | 72 | Stage II |
| DO48721 | 900 | alive | FEMALE | 58 | Stage I |
| DO45235 | 900 | alive | FEMALE | 68 | Stage I |
| DO45251 | 900 | alive | FEMALE | 57 | Stage II |
| DO48692 | 900 | alive | FEMALE | 77 | Stage III |
| DO23531 | 900 | alive | FEMALE | 78 | Stage III |
| DO45201 | 900 | alive | MALE | 49 | Stage I |
| DO48709 | 900 | alive | MALE | 74 | Stage I |
| DO48681 | 900 | alive | MALE | 57 | Stage II |
| DO45237 | 900 | alive | MALE | 58 | Stage II |
| DO23533 | 900 | alive | MALE | 61 | Stage II |
| DO48689 | 900 | alive | MALE | 63 | Stage II |
| DO48693 | 900 | alive | MALE | 71 | Stage II |
| DO48691 | 900 | alive | MALE | 71 | Stage II |
| DO23537 | 900 | death | MALE | 79 | Stage II |
| DO45233 | 900 | alive | MALE | 83 | Stage III |
| DO45243 | 930 | alive | FEMALE | 74 | Stage III |
| DO50802 | 930 | alive | MALE | 49 | Stage II |
| DO23536 | 930 | alive | MALE | 56 | Stage II |
| DO23541 | 930 | alive | MALE | 65 | Stage II |
| DO45231 | 930 | alive | MALE | 84 | Stage II |
| DO23529 | 930 | alive | MALE | 72 | Stage IV |
| DO23532 | 960 | alive | FEMALE | 71 | Stage I |
| DO23535 | 960 | alive | MALE | 67 | Stage I |
| DO23544 | 960 | alive | MALE | 67 | Stage II |
| DO45163 | 960 | alive | MALE | 76 | Stage II |
| DO23542 | 960 | alive | MALE | 66 | Stage III |
| DO45303 | 990 | alive | FEMALE | 62 | Stage III |
| DO23550 | 990 | alive | MALE | 69 | Stage I |
| DO45229 | 990 | alive | MALE | 74 | Stage I |
| DO45261 | 990 | alive | MALE | 74 | Stage III |
| DO23549 | 990 | alive | MALE | 74 | Stage III |
| DO48686 | 990 | alive | MALE | 75 | Stage III |
| DO23528 | 1020 | alive | FEMALE | 45 | Stage II |
| DO45227 | 1020 | alive | FEMALE | 57 | Stage II |
| DO23547 | 1020 | alive | MALE | 74 | Stage I |
| DO23543 | 1020 | alive | MALE | 63 | Stage II |
| DO45265 | 1020 | alive | MALE | 73 | Stage III |
| DO45223 | 1020 | alive | MALE | 67 | Stage IV |
| DO23524 | 1050 | alive | FEMALE | 71 | Stage II |
| DO45207 | 1050 | alive | MALE | 68 | Stage I |
| DO45225 | 1050 | alive | MALE | 69 | Stage I |
| DO23527 | 1050 | alive | MALE | 72 | Stage I |
| DO23546 | 1050 | alive | MALE | 63 | Stage II |
| DO23526 | 1050 | alive | MALE | 66 | Stage III |
| DO23525 | 1050 | alive | MALE | 71 | Stage IV |
| DO45241 | 1080 | alive | MALE | 49 | Stage I |
| DO45219 | 1080 | alive | MALE | 78 | Stage II |
| DO23523 | 1080 | alive | MALE | 52 | Stage III |
| DO45117 | 1110 | death | FEMALE | 73 | Stage II |
| DO23539 | 1110 | alive | MALE | 77 | Stage I |
| DO23521 | 1110 | alive | MALE | 63 | Stage III |
| DO45175 | 1140 | alive | MALE | 74 | Stage II |
| DO48677 | 1140 | alive | MALE | 58 | Stage III |
| DO45203 | 1170 | alive | FEMALE | 71 | Stage II |
| DO45197 | 1170 | alive | MALE | 51 | Stage II |
| DO45215 | 1170 | alive | MALE | 65 | Stage II |
| DO45173 | 1170 | alive | MALE | 63 | Stage III |
| DO45191 | 1200 | alive | FEMALE | 61 | Stage II |
| DO45181 | 1200 | alive | FEMALE | 84 | Stage II |
| DO45189 | 1200 | alive | FEMALE | 85 | Stage II |
| DO45213 | 1200 | alive | MALE | 58 | Stage I |
| DO23517 | 1200 | alive | MALE | 62 | Stage II |
| DO48679 | 1200 | alive | MALE | 72 | Stage II |
| DO23513 | 1200 | alive | MALE | 76 | Stage III |
| DO45092 | 1200 | death | MALE | 62 | Stage IV |
| DO23515 | 1230 | alive | MALE | 71 | Stage II |
| DO45151 | 1230 | alive | MALE | 64 | Stage III |
| DO45187 | 1260 | alive | FEMALE | 32 | Stage I |
| DO45149 | 1260 | alive | FEMALE | 70 | Stage II |
| DO45177 | 1260 | alive | FEMALE | 81 | Stage III |
| DO45297 | 1260 | death | FEMALE | 61 | Stage IV |
| DO23512 | 1260 | alive | MALE | 60 | Stage I |
| DO227643 | 1260 | alive | MALE | 79 | Stage II |
| DO48674 | 1260 | alive | MALE | 80 | Stage II |
| DO23514 | 1290 | alive | MALE | 64 | Stage II |
| DO45096 | 1290 | death | MALE | 69 | Stage II |
| DO45279 | 1290 | alive | MALE | 67 | Stage III |
| DO45143 | 1290 | alive | MALE | 69 | Stage III |
| DO23522 | 1320 | alive | FEMALE | 82 | Stage I |
| DO45137 | 1320 | alive | MALE | 38 | Stage I |
| DO45091 | 1320 | death | MALE | 56 | Stage II |
| DO23518 | 1320 | alive | MALE | 67 | Stage II |
| DO45141 | 1320 | alive | MALE | 73 | Stage II |
| DO45139 | 1320 | alive | MALE | 63 | Stage III |
| DO45199 | 1350 | alive | FEMALE | 86 | Stage II |
| DO45169 | 1350 | alive | MALE | 74 | Stage III |
| DO45209 | 1350 | alive | MALE | 70 | Stage IV |
| DO45153 | 1380 | alive | FEMALE | 77 | Stage II |
| DO45135 | 1410 | alive | MALE | 77 | Stage I |
| DO23520 | 1410 | alive | MALE | 59 | Stage IV |
| DO45299 | 1440 | alive | FEMALE | 70 | Stage I |
| DO45301 | 1440 | death | MALE | 56 | Stage II |
| DO45167 | 1440 | death | MALE | 67 | Stage II |
| DO45129 | 1470 | alive | MALE | 61 | Stage III |
| DO45159 | 1470 | alive | MALE | 65 | Stage III |
| DO45121 | 1530 | alive | MALE | 61 | Stage III |
| DO45113 | 1560 | alive | MALE | 58 | Stage III |
| DO45109 | 1590 | alive | MALE | 63 | Stage II |
| DO45111 | 1590 | alive | MALE | 74 | Stage II |
| DO45099 | 1590 | alive | MALE | 46 | Stage III |
| DO45095 | 1620 | alive | MALE | 66 | Stage II |
| DO45097 | 1620 | alive | MALE | 69 | Stage III |
| DO45094 | 1650 | alive | MALE | 76 | Stage III |
| DO45105 | 1680 | alive | MALE | 61 | Stage I |
| DO45101 | 1680 | alive | MALE | 63 | Stage II |
| DO45125 | 1680 | alive | MALE | 58 | Stage III |
| DO45107 | 1710 | alive | FEMALE | 63 | Stage II |
| DO45157 | 1980 | alive | MALE | 62 | Stage II |
| DO45155 | 2160 | alive | MALE | 69 | Stage II |

Table S3. The primers’ sequences for qRT-PCR analysis.

| Primer | Sequences |
| --- | --- |
| METTL3 | F: 5’-AGATGGGGTAGAAAGCCTCCT-3’ |
|  | R:5’-TGGTCAGCATAGGTTACAAGAGT-3’ |
| NSUN4 | F: 5’-CTGTCTCCATCCTGTTCGTG-3’ |
|  | R:5’-CTGCTCACTGGCTTCTCCTT-3’ |
| TRMT6 | F: 5’-GGAATGCTACACAAAACTGC-3’ |
|  | R:5’-AGAAGATAACCCCCACCTC-3’ |
| YTHDF1 | F: 5’-GCAAGGAACGGCAGAGT-3’ |
|  | R:5’-CAACGACAAGAAAGGCAAAGAT-3’ |
